# Stabilin-2 modulates the efficiency of myoblast fusion during myogenic differentiation and muscle regeneration

**DOI:** 10.1038/ncomms10871

**Published:** 2016-03-14

**Authors:** Seung-Yoon Park, Youngeun Yun, Jung-Suk Lim, Mi-Jin Kim, Sang-Yeob Kim, Jung-Eun Kim, In-San Kim

**Affiliations:** 1 Department of Biochemistry, School of Medicine, Dongguk University, Gyeongju 780-714, Republic of Korea; 2 Department of Biochemistry and Cell Biology, School of Medicine, Kyungpook National University, Daegu 700-422, Republic of Korea; 3 Department of Convergence Medicine, University of Ulsan, College of Medicine & Asan Institute for Life Sciences, Asan Medical Center, Seoul 138-736, Republic of Korea; 4 Department of Molecular Medicine, School of Medicine, Kyungpook National University, Daegu 700-422, Republic of Korea; 5 Biomedical Research Institute, Korea Institute Science and Technology, Seoul 136-791, Republic of Korea; 6 KU-KIST school, Korea University, Seoul 136-701, Republic of Korea

## Abstract

Myoblast fusion is essential for the formation of skeletal muscle myofibres. Studies have shown that phosphatidylserine is necessary for myoblast fusion, but the underlying mechanism is not known. Here we show that the phosphatidylserine receptor stabilin-2 acts as a membrane protein for myoblast fusion during myogenic differentiation and muscle regeneration. Stabilin-2 expression is induced during myogenic differentiation, and is regulated by calcineurin/NFAT signalling in myoblasts. Forced expression of stabilin-2 in myoblasts is associated with increased myotube formation, whereas deficiency of stabilin-2 results in the formation of small, thin myotubes. Stab2-deficient mice have myofibres with small cross-sectional area and few myonuclei and impaired muscle regeneration after injury. Importantly, myoblasts lacking stabilin-2 have reduced phosphatidylserine-dependent fusion. Collectively, our results show that stabilin-2 contributes to phosphatidylserine-dependent myoblast fusion and provide new insights into the molecular mechanism by which phosphatidylserine mediates myoblast fusion during muscle growth and regeneration.

## Abstract (web summary)

Phosphatidylserine and its receptors are associated with cell-cell fusion. Here, the authors show the phosphatidylserine receptor stabilin-2 is expressed by muscle cells and plays a vital role in myoblast fusion and post-injury muscle regeneration in mice.

## Graphical abstract

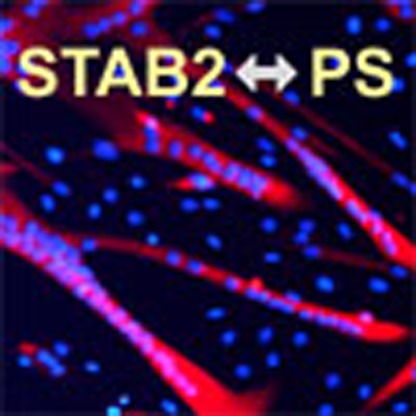

Skeletal muscle consists of multinucleated myofibres that form through the fusion of mononucleated myoblasts. This process is required for skeletal muscle formation during myogenesis and post-injury regeneration and growth. Myoblast fusion follows an ordered set of cellular events that include cell migration, alignment, adhesion and membrane fusion^1^,^2^. Many molecules are believed to participate in myoblast fusion and muscle regeneration, including various secreted proteins, membrane receptors and transcription factors. However, the precise mechanisms by which myoblasts fuse to form multinucleated cells are unknown.

Phosphatidylserine exists in the inner leaflet of the plasma membrane and is externalized during apoptosis; however, phosphatidylserine exposure on the cell surface also occurs in non-apoptotic cells during various cellular processes^3^,^4^. Several lines of evidence indicate that phosphatidylserine has important roles in various cell–cell fusion processes, including myoblast fusion. For example, phosphatidylserine is exposed at the cell surface of viable myoblasts in developing skeletal muscles^5^, suggesting that it may function in the differentiation of myoblasts. Consistent with this idea, phosphatidylserine is transiently exposed at cell–cell contact regions during myogenic differentiation, and blockade of phosphatidylserine on the cell surface (using the phosphatidylserine-binding protein, annexin V) abrogates myotube formation^6^. Anti-phosphatidylserine antibody-mediated masking of phosphatidylserine inhibits myoblast fusion during myogenic differentiation^7^, and phosphatidylserine is implicated in other fusion models, including syncytiotrophoblast formation and macrophage fusion. For example, an efflux of phosphatidylserine is associated with intercellular cytotrophoblast fusion, and a monoclonal anti-phosphatidylserine antibody inhibits the formation of syncytiotrophoblasts^8^,^9^. In the context of macrophages, exposure and recognition of phosphatidylserine is required for polykaryon formation^10^. These findings imply that phosphatidylserine-dependent fusion is a mechanism in various fusion models.

Three representative phosphatidylserine receptors have been identified as being involved in recognizing phosphatidylserine on the surface of apoptotic cells: Tim4, Bai1 and stabilin-2 (Stab2) (refs 11, 12, 13). The recognition of cell-surface phosphatidylserine by phosphatidylserine receptors induces intracellular signalling via the CrkII/Dock180/ELMO or Gulp1 pathways^14^,^15^, which converge at CED-10/Rac1 to mediate actin rearrangement and subsequent engulfment of cell corpses^16^. The GTPase, Rac1, is required for cytoskeletal rearrangement during myoblast fusion, in a role that is conserved from flies to mice^17^,^18^,^19^. These observations raise the possibility that phosphatidylserine receptors are involved in both cell–cell fusion and apoptotic cell clearance. Indeed, activation of Bai1 signalling by apoptotic myoblasts has been shown to promote fusion between healthy myoblasts^20^. However, in this study apoptotic myoblasts did not directly fuse with the healthy myoblasts^20^. Although phosphatidylserine was externalized to the cell surface in myoblasts fusing into myotubes, these cells were not undergoing apoptosis^6^. Thus, the molecular mechanism through which cell-surface-exposed phosphatidylserine mediates the fusion of viable myoblasts during myogenic differentiation is unknown.

Stab2 is a type I transmembrane receptor that contributes to multiple processes, including endocytosis^21^,^22^,^23^,^24^, cell–cell interactions^25^,^26^ and apoptotic and necrotic cell clearance^13^,^27^. It is expressed in the sinusoidal endothelial cells of spleen, liver, lymph node and bone marrow, as well as in some populations of macrophages^13^,^28^, but its expression pattern in other tissues is not defined. Stab2 acts as a phosphatidylserine receptor, mediating both the clearance of cell corpses in macrophages^13^ and the capture of phosphatidylserine-exposed red blood cells by hepatic sinusoidal endothelial cells^29^. Atypical epidermal growth factor-like (EGF-like) domains in the four EGF-like domain repeats of Stab2 specifically bind phosphatidylserine^30^.

Here we report for the first time that Stab2 is expressed in muscle tissues and myoblasts. We show that Stab2 deficiency results in the formation of small and thin myotubes *in vitro* and impairs post-injury muscle regeneration *in vivo*. We also show that Stab2 contributes to phosphatidylserine-dependent fusion during myogenic differentiation. Our data collectively reveal a function for Stab2 during myoblast fusion, and provide new insights into the molecular mechanism by which phosphatidylserine mediates myoblast fusion.

## Results

### Stab2 expression in skeletal muscles and myoblasts

**Figure 1:**
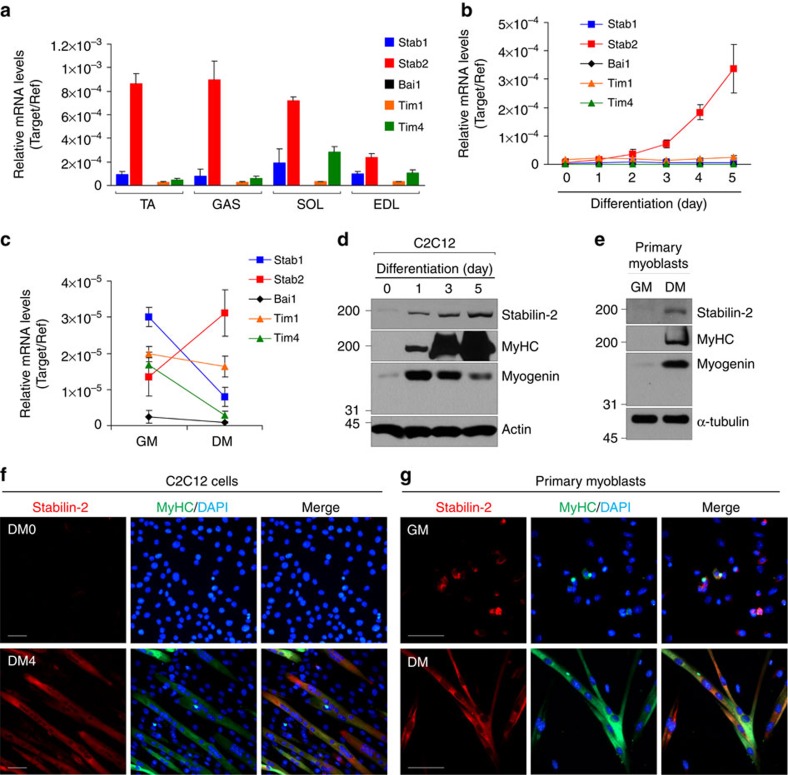
Stabilin-2 expression in muscle tissues and during myogenic differentiation. (**a**) The expressions of PS-recognizing receptors (Tim1, Tim4, Bai1, stabilin-1 and stabilin-2) were examined in mouse skeletal muscles by quantitative real-time PCR. Data are presented as mean±s.d. of at least three independent experiments. (**b**,**c**) Expression levels of PS-recognizing receptors were examined in C2C12 cells (**b**) and primary myoblasts (**c**) during myogenic differentiation by quantitative real-time PCR. Data are presented as mean±s.d. of at least three independent experiments. GM, growth medium; DM, differentiation medium. (**d**,**e**) Expression levels of stabilin-2 protein were determined in C2C12 cells (**d**) and primary myoblasts (**e**) during myogenic differentiation by Western blotting. Representative results from three independent experiments are shown. MyHC, myosin heavy chain. Full-size blots are shown in Supplementary Fig. 15. (**f**,**g**) C2C12 cells (**f**) and primary myoblasts (**g**) were induced to differentiate in differentiation medium (DM), and the expressions of stabilin-2 and myosin heavy chain (MyHC) were analysed at the indicated times by immunostaining. Scale bar, 50 μm.

Several molecules have been identified as phosphatidylserine-specific receptors, including Tim4, Bai1 and Stab2 (refs 11, 12, 13). Here we used quantitative real-time PCR (qrtPCR) to determine the expression levels of these phosphatidylserine receptors in various mouse skeletal muscles, including the tibialis anterior, extensor distalis longus, gastrocnemius and soleus muscles. We found that Stab2 was the main phosphatidylserine receptor expressed in these skeletal muscles, with relatively lower expression detected for Tim4 and only rare expression of Bai1 in the tested muscle tissues (Fig. 1a). Next, we analysed the expression levels of these receptors during myogenic differentiation. We found that Stab2 mRNA expression was upregulated in C2C12 cells undergoing myogenic differentiation, whereas the mRNAs encoding Tim4 and Bai1 were barely detectable in both proliferating and differentiating C2C12 cells (Fig. 1b). Low expression of stabilin-1 (ref. 31) and Tim1 (ref. 32), which are known as receptors for phosphatidylserine recognition among other family of Stab2 and Tim4, respectively, were detected in skeletal muscles and C2C12 cells (Fig. 1a,b). The Stab2 mRNA was increased in primary myoblasts at 30 h after the induction of differentiation (Fig. 1c). In contrast, Tim4 and stabilin-1 mRNA expression was declined after the induction of differentiation (Fig. 1c). Consistent with the results of our mRNA expression analysis, Stab2 protein expression was also increased in C2C12 cells and primary myoblasts after the induction of differentiation (Fig. 1d,e). To further examine the expression of Stab2 during myogenic differentiation, we performed immunofluorescent staining during differentiation in C2C12 cells and primary myoblasts. We found that Stab2 was only expressed in <15% of proliferating myoblasts, but it was highly expressed in myotubes, where it colocalized with myosin heavy chain (MyHC), which is a marker of myogenic differentiation (Fig. 1f,g). These results suggest that the phosphatidylserine receptor Stab2 may be involved in the fusion of myoblasts during myogenic differentiation.

### Stab2 expression is regulated by NFATc1

**Figure 2:**
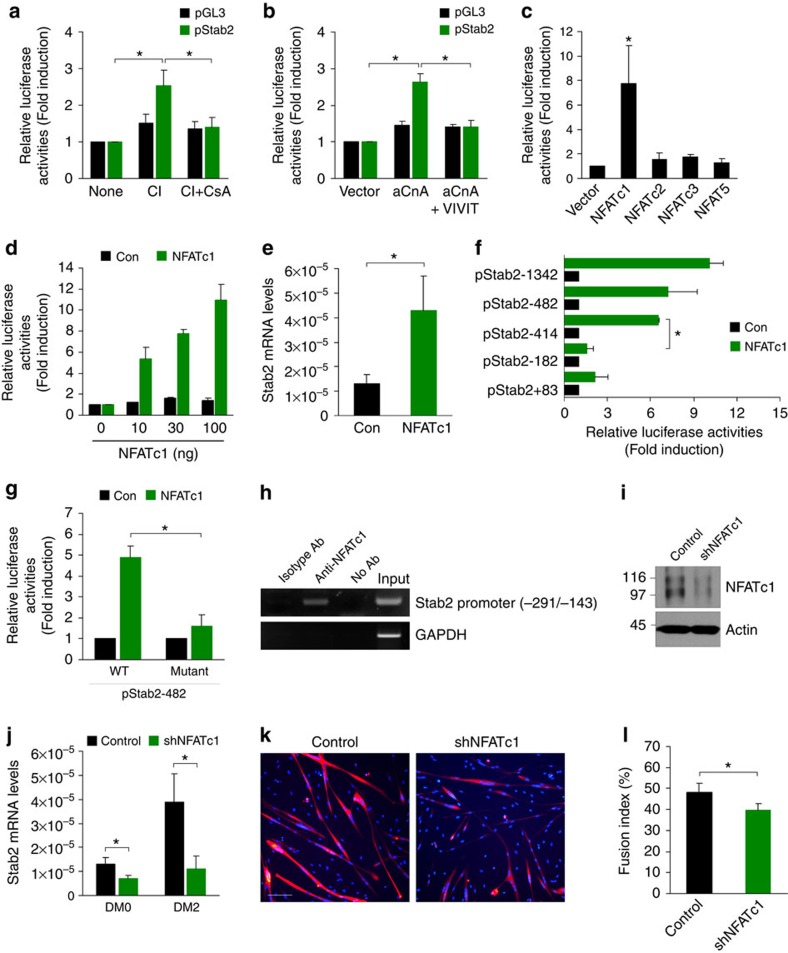
Stabilin-2 expression is regulated by calcineurin/NFAT signalling. (**a**) Luciferase assay in primary myoblasts transfected with the stabilin-2 promoter construct. At 24 h post-transfection, cells were incubated with vehicle, calcium ionophore A23187 (CI, 1 μM), or CI plus cyclosporine A (CsA, 1 μM) for 24 h. (**b**) Luciferase assay in primary myoblasts transfected with the stabilin-2 promoter construct along with plasmid encoding activated calcineurin (aCnA) and/or GFP-VIVIT. (**c**) Luciferase assay in primary myoblasts transfected with stabilin-2 promoter construct along with the indicated NFAT expression vector. (**d**) Luciferase assay in primary myoblasts transfected with the stabilin-2 promoter construct along with the indicated amount of plasmid encoding NFATc1. (**e**) Real-time PCR analysis of Stab2 mRNA in primary myoblasts infected with retrovirus encoding NFATc1 or retrovirus from pMXs IRES-puro vector. (**f**) Luciferase assay in primary myoblasts transfected the stabilin-2 promoter construct or a series of 5' deletion constructs along wth plasmid encoding NFATc1 or empty vector (Con). (**g**) Luciferase assay in primary myoblasts transfected with the stabilin-2 promoter construct (nt −482 to +205) or its NFAT mutant along with plasmid encoding NFATc1. (**h**) Soluble chromatin was prepared from human myoblasts (DM1) for ChIP assays and immunoprecipitated with anti-NFATc1 antibody. An isotype-matched control antibody was used as the negative control. Immunoprecipitates were subjected to PCR with primers specific to the NFAT-responsive element in stabilin-2 promoter. GAPDH primers were used as a negative control. The result shown is a representative of three independent experiments. (**i**) Representative immunoblot of NFATc1 in primary myoblasts infected with retrovirus encoding NFATc1 shRNA or retrovirus from pMXs-U6 vector. (**j**–**l**) Primary myoblasts infected with retrovirus encoding NFATc1 shRNA (shNFATc1) or retrovirus from pMXs-U6 vector (Control) were induced to differentiate for 2 days. Expression of stabilin-2 mRNA was analysed by quantitative real-time PCR (**j**). Representative images in DM2 (**k**) are shown. Scale bars, 100 μm. Fusion indices (**l**) were calculated. Relative luciferase activities were normalized as fold over that of the stabilin-2 promoter in the absence of NFATc1. Data are presented as mean±s.d. of three independent experiments. Asterisks indicate statistical significance (*P<0.05, Student's *t*-test).

To explore the molecular mechanism responsible for the expression of Stab2 in skeletal muscle, we determined the transcription start site of the Stab2 gene using 5′ rapid amplification of cDNA ends (RACE) PCR (Supplementary Fig. 1) and generated a Stab2 promoter construct (pStab2-Luc) consisting of ∼1,500 bp (nucleotides −1,342 to +205). Sequence analysis revealed that there are several *cis*-responsive elements for the nuclear factor of activated T cells (NFAT) family of transcription factors in the Stab2 promoter region (Supplementary Fig. 2). NFAT is one downstream target of calcium-dependent calcineurin signalling^33^, which regulates many aspects of skeletal myogenesis. Activation of NFAT via calcineurin-dependent signalling regulates the expression of skeletal muscle-specific genes that are associated with myogenic differentiation^34^,^35^. We determined whether calcium-dependent calcineurin signalling affects the expression of Stab2 in muscle cells. As shown in Fig. 2a and Supplementary Fig. 3a, treatment with the calcium ionophore A23187 caused a 2.5-fold and 6.5-fold increase in Stab2 promoter activity in primary myoblasts and C2C12 cells, respectively, and this effect was robustly abrogated by co-treatment with the calcineurin inhibitor, cyclosporine A (CsA). Furthermore, Stab2 promoter activity was increased by cotransfection of activated calcineurin, and this increase was robustly abrogated by co-expression of green fluorescent protein-fusion VIVIT, a specific inhibitor of NFAT activation^36^ (Fig. 2b and Supplementary Fig. 3b). Next, we examined the promoter activity of Stab2 in the presence of various NFAT isoforms in myoblasts. Our results revealed that NFATc1 increased the promoter activity of Stab2 in primary myoblasts and C2C12 cells by 7.7-fold and 11.9-fold, respectively, whereas NFATc3 upregulates Stab2 promoter activity in only C2C12 cells (Fig. 2c and Supplementary Fig. 3c). NFATc2 and NFAT5 had no such effect (Fig. 2c). Furthermore, NFATc1 increased the promoter activity of Stab2 in a dose-dependent manner (Fig. 2d and Supplementary Fig. 3d) and enhanced the expression of the Stab2 mRNA (Fig. 2e and Supplementary Fig. 3e). There are five putative NFAT-binding motifs in the 5′ flanking region of the human Stab2 promoter (Supplementary Fig. 2b). To assess whether any of these regions acts as a *cis*-responsive element for NFATc1, we generated a series of 5′ deletion constructs of the Stab2 promoter (Supplementary Fig. 2b) and analysed promoter activity in the presence of NFATc1. We found that deletion of nucleotides −414 to −182 significantly decreased the Stab2 promoter activity induced by NFATc1 (Fig. 2f and Supplementary Fig. 3f), suggesting that the putative NFAT-responsive element at nucleotides −188 to −183 is important for Stab2 expression. Indeed, mutation in this NFAT-binding motif abolished the NFATc1-mediated activation of the Stab2 promoter in primary myoblasts and C2C12 cells (Fig. 2g and Supplementary Fig. 3g). We found that the amount of NFATc1 in the nucleus was increased after induction of differentiation (Supplementary Fig. 4a). To further determine whether the putative NFAT-binding motif at nucleotides −188 to −183 is a functional binding site for the NFATc1 proteins, we performed a promoter enzyme immunoassay using nuclear extracts from 293FT cells transfected with plasmids encoding NFATc1. Our results revealed that the nuclear-localized NFATc1 proteins bound to oligonucleotides containing the NFAT-binding motif, but not mutant NFAT-binding motif (Supplementary Fig. 4b). Consistent with these findings, electrophoretic mobility shift assay (EMSA) verified the formation of a DNA-protein complex between an oligonucleotide flanking the NFAT-binding motif at nucleotides −188 to −183 and nuclear NFAT proteins (Supplementary Fig. 4c). To examine whether nuclear NFATc1 binds directly to the NFAT-responsive element *in vivo*, we performed chromatin immunoprecipitation (ChIP) analyses in human myoblasts. Our results revealed that an anti-NFATc1 antibody, but not isotype-matched IgG, precipitated a DNA fragment encompassing the NFAT-binding motif (Fig. 2h). As a negative control, no PCR signal was observed for GAPDH. To assess whether Stab2 expression by NFATc1 is required for myoblast fusion during myogenic differentiation, we performed NFATc1 knockdown in primary myoblasts using retroviral infection (Fig. 2i). Downregulation of NFATc1 decreased the expression of Stab2 mRNA (Fig. 2j) and myoblast fusion during myogenic differentiation (Fig. 2k,l). Similar results were obtained with C2C12 cells (Supplementary Fig. 5). Taken together, these results demonstrate that Stab2 is a direct transcriptional target of NFATc1 in muscle cells.

### Stab2 promotes myoblast fusion

**Figure 3:**
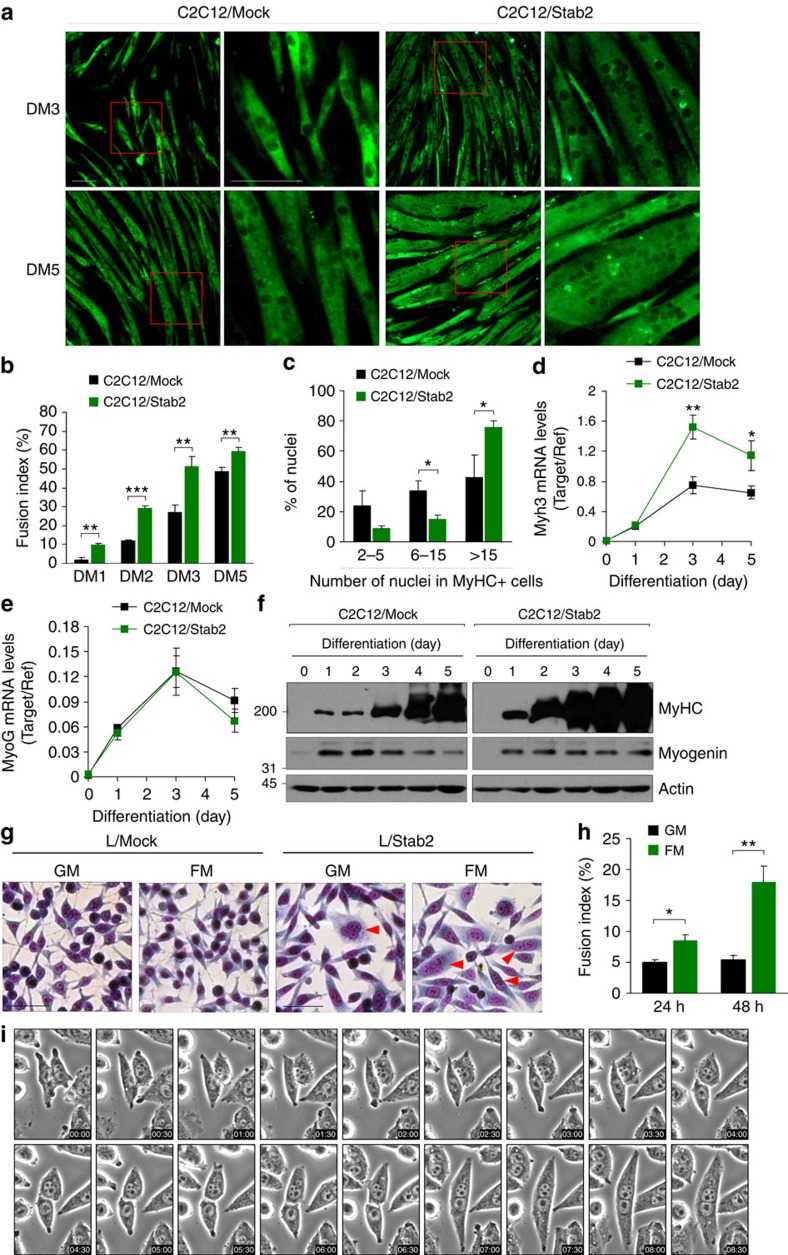
The overexpression of Stabilin-2 enhances myotube formation in myoblasts. (**a**) C2C12/Stab2 and C2C12/Mock cells were induced to differentiate for the indicated times. Cells were then fixed and immunostained with anti-MyHC antibody. Representative microscopic fields are shown. Scale bar, 100 μm. Red boxes are shown at higher magnification. (**b**) C2C12/Stab2 and C2C12/Mock cells were induced to differentiate for the indicated times, and fusion indices were calculated. Data are presented as mean±s.d. of three independent experiments. (**c**) After 5 days of differentiation (DM5), the percentage of nuclei present in MyHC-positive myotubes with the indicated number of nuclei were quantified in C2C12/Stab2 and C2C12/Mock cells. Data are presented as mean±s.d. of three independent experiments. (**d**,**e**) C2C12/Stab2 and C2C12/Mock cells were induced to differentiate for the indicated times. The levels of embryonic MyHC (Myf3, **d**) and myogenin (MyoG, **e**) mRNA were analysed by quantitative real-time PCR. Data are presented as mean±s.d. of three independent experiments. (**f**) The levels of MyHC and myogenin proteins were analysed in C2C12/Stab2 and C2C12/Mock cells during differentiation by immunoblotting. Full-size blots are shown in Supplementary Fig. 15. (**g**,**h**) L cells stably transfected with stabilin-2 expression vector (L/Stab2) or empty vector (L/Mock) were incubated in growth medium (GM) or fusion medium (FM). Representative images (**g**) are shown. Scale bars, 100 μm. Fusion indices of stabilin-2-expressing cells at the indicated time points are shown in graph (**h**). Data are presented as mean±s.d. of at least three independent experiments. (**i**) Differential interference contrast (DIC) images from Supplementary Movie 1 showed cell–cell fusion in stabilin-2-expressing cells. Asterisks indicate statistical significance (*P<0.05, **P<0.01, ***P<0.001, Student's *t*-test).

To further determine the role of Stab2 in myoblast fusion, we generated C2C12 myoblasts expressing FLAG-tagged human Stab2 (C2C12/Stab2) (Supplementary Fig. 6a,b) and analysed myotube formation during differentiation. Forced expression of Stab2 in C2C12 myoblasts promoted muscle cell fusion and generated larger myotubes with more nuclei, compared with control C2C12 cells (C2C12/Mock) (Fig. 3a and Supplementary Fig. 6c). To quantify myoblast fusion, we calculated the fusion index by expressing the number of nuclei within MyHC-positive myotubes with ≥2 nuclei as a percentage of the total nuclei. At all time points after induction of differentiation, the fusion index was significantly higher in C2C12/Stab2 cells compared with C2C12/Mock cells (Fig. 3b). Nuclear number analysis revealed that the percentage of the nuclei in large myotubes (with >15 nuclei) was significantly higher in C2C12/Stab2 cells compared with C2C12/Mock cells at DM5 (Fig. 3c), suggesting that Stab2 is involved in myonuclear accretion to promote myotube formation. Consistent with the results of our fusion analysis, embryonic MyHC (eMyHC) mRNA, which is expressed in newly formed myotubes during differentiation, was more highly expressed in C2C12/Stab2 cells versus C2C12/Mock cells (Fig. 3d,f). However, no difference was observed in myogenin expression in both cells (Fig. 3e,f), indicating that myogenic commitment were not affected by Stab2 expression. To directly examine the role of Stab2 as a membrane protein for cell–cell fusion, we analysed whether overexpression of Stab2 could induce cell fusion in L cells, a fibroblast cell line that lacks fusogenic activity. In growth medium, a few multinucleated cells were observed in Stab2-overexpressing cell (L/Stab2) cultures but not L/Mock cell cultures (Fig. 3g). Given the role of Stab2 as a phosphatidylserine receptor in apoptotic cell clearance, it is possible that an interaction between Stab2 and phosphatidylserine could induce cell fusion. Because the interaction between Stab2 and phosphatidylserine was previously shown to be enhanced under low-pH conditions^37^, we analysed the effect of low-pH fusion medium on the formation of multinucleated cells by cultured fibroblasts. Indeed, the formation of multinucleated cells in L/Stab2 cultures was increased in low-pH fusion medium (Fig. 3g,h). Real-time videomicroscopic analyses verified that the multinucleated cells in L/Stab2 cultures were formed by cell–cell fusion (Fig. 3i and Supplementary Video 1). Collectively, these results suggest that Stab2 can enhance myotube formation via increased myoblast fusion.

### Myofibre CSA is decreased in *Stab2* ^*−/−*^ skeletal muscle

**Figure 4:**
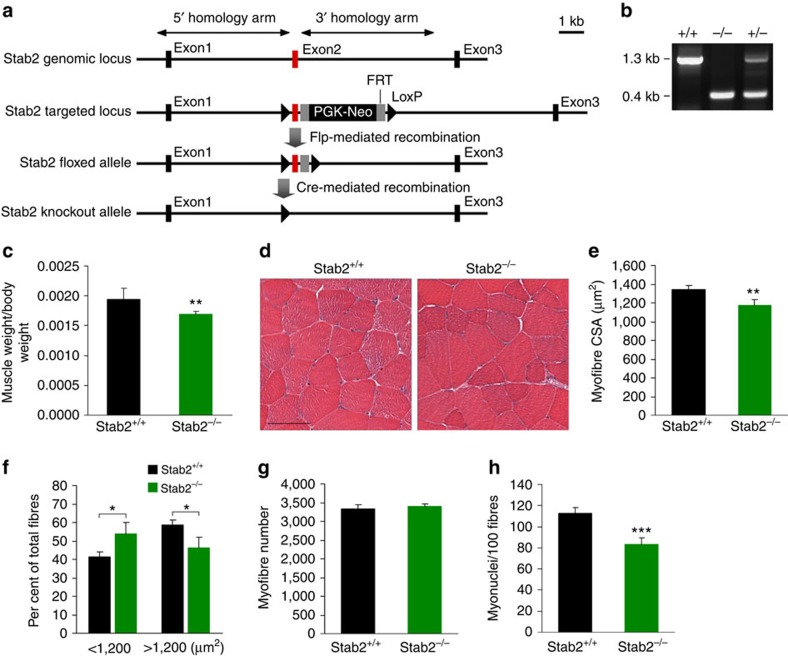
Myofibre CSAs and myonuclear numbers are decreased in *Stab2*^*−/−*^ muscles. (**a**) Targeting of the *Stabilin-2* gene. The two *lox*P sites flanking the exon 2 (red) of the *Stab2* gene are represented as triangles. Cre-mediated deletion of Stabilin-2 exon 2 (red) was achieved by crossing heterozygous *Stab2*^*+/flox*^ mice with C57BL/6 Cre-deleter expressing constitutively recombinase Cre. (**b**) PCR analysis of genomic DNA clearly discriminated the *Stab2*^*+/+*^, *Stab2*^*+/−*^ and *Stab2*^*−/−*^ genotypes. (**c**) Tibialis anterior (TA) muscles were isolated from 9-week-old male *Stab2*^*+/+*^ and *Stab2*^*−/−*^ mice, and muscle weight relative to body weight was analysed. Data are presented as mean±s.d. (*n*=8) of each genotype. (**d**) Representative sections of *Stab2*^*+/+*^ and *Stab2*^*−/−*^ TA muscles stained with H&E. Scale bar, 50 μm. (**e**) Cross-sectional areas (CSAs) of myofibres from *Stab2*^*+/+*^ and *Stab2*^*−/−*^ TA muscles. Data are presented as mean±s.d. (*n*=4) of each genotype. (**f**) The distribution of small and large myofibres from TA muscles of 9-week-old male *Stab2*^*+/+*^ and *Stab2*^*−/−*^ mice. Data are presented as mean±s.d. (*n*=4) of each genotype. (**g**) Myofibre numbers were analysed in TA muscles from 9-week-old male *Stab2*^*+/+*^ and *Stab2*^*−/−*^ mice. Data are presented as mean±s.d. (*n*=4) of each genotype. (**h**) DAPI-stained nuclei within dystrophin-positive sarcolemma were analysed in TA muscles from *Stab2*^*+/+*^ and *Stab2*^*−/−*^ mice, and numbers of myonuclei per 100 fibres were calculated. Data are presented as mean±s.d. (*n*=5) of each genotype. Asterisks indicate statistical significance (*P<0.05, **P<0.01, ***P<0.001, Student's *t*-test).

On the basis of the formation of large myotubes following forced expression of Stab2 (Fig. 3a), we hypothesized that Stab2 may modulate the efficiency of myoblast fusion by promoting myonuclear accretion. To assess the function of the *Stab2* gene in the skeletal muscles of mice, we generated a null allele by deleting the second exon of the *Stab2* gene (Fig. 4a). Deletion of exon 2 of *Stab2* gene was confirmed by PCR analysis of genomic DNA and mRNA (Fig. 4b and Supplementary Fig. 7a,b). Ablation of Stab2 protein was also confirmed by immunoblotting (Supplementary Fig. 7d,e). Stab2 deficiency had no effect on the expression of stabilin-1, which shares common features with Stab2 (Supplementary Fig. 7c–e). Bai1 and Tim4 proteins were not detected in *Stab2*^*+/+*^ and *Stab2*^*−/−*^ tibialis anterior muscles (Supplementary Fig. 7f). Our examination of skeletal muscles revealed that the tibialis anterior muscle weight related to body weight in 9-week-old male *Stab2*^*−/−*^ mice was 12.6% lower than that of *Stab2*^*+/+*^ mice (Fig. 4c). To test whether this decrease in muscle weight reflected reductions in the size or number of individual myofibres, we examined the cross-sectional area (CSA) and number of myofibres in tibialis anterior muscles from *Stab2*^*+/+*^ and *Stab2*^*−/−*^ mice. Our results showed that the CSA of *Stab2*^*−/−*^ myofibres was significantly smaller than that of *Stab2*^*+/+*^ myofibres (Fig. 4d,e). *Stab2*^*−/−*^ tibialis anterior muscles contained more small myofibres (<1,200 μm^2^) and fewer large myofibres (>1,200 μm^2^) compared with *Stab2*^*+/+*^ tibialis anterior muscles (Fig. 4f), but the number of myofibres did not significantly differ between *Stab2*^*−/−*^ and *Stab2*^*+/+*^ tibialis anterior muscles (Fig. 4g). These findings indicated that the reduced *Stab2*^*−/−*^ tibialis anterior muscle weight reflects a decrease in myofibre size rather than a change in the number of myofibres. The reduced CSA in *Stab2*^*−/−*^ tibialis anterior muscles suggests that *Stab2*^*−/−*^ myofibres may also have reduced myonuclei number due to decreased myoblast fusion. To determine whether myonuclei number is reduced in *Stab2*^*−/−*^ myofibres, we analysed the numbers of DAPI-stained myonuclei inside of the dystrophin-stained sarcolemma in cross and longitudinal sections of *Stab2*^*−/−*^ and *Stab2*^*+/+*^ tibialis anterior muscles. Our results revealed that *Stab2*^*−/−*^ myofibres contained fewer myonuclei than *Stab2*^*+/+*^ myofibres (Fig. 4h and Supplementary Fig. 8), suggesting that the reduced CSA of *Stab2*^*−/−*^ muscle is due to decreased myoblast fusion.

### Myoblast fusion is impaired in Stab2-deficient myoblasts

**Figure 5:**
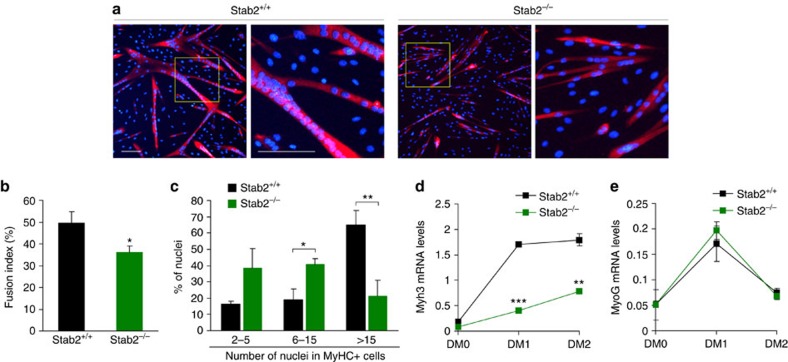
Myoblast fusion is impaired in Stab2-deficient myoblasts. (**a**) Myoblasts isolated from *Stab2*^*+/+*^ and *Stab2*^*−/−*^ mice were plated at equal densities and induced to differentiate. After 48 h, cells were fixed and immunostained with anti-MyHC antibody. Representative microscopic fields are presented. Yellow boxes are shown at higher magnification. Scale bar, 100 μm. (**b**) After 2 days of differentiation (DM2), fusion indices were determined for *Stab2*^*+/+*^ and *Stab2*^*−/−*^ myoblasts. Data are presented as mean±s.d. of at least three independent experiments. (**c**) *Stab2*^*+/+*^ and *Stab2*^*−/−*^ myoblasts were fixed and immunostained for anti-MyHC antibody at DM2, and then the percentage of nuclei present in MyHC-positive cells with the indicated number of nuclei were calculated. Data are presented as mean±s.d. of at least three independent experiments. (**d**,**e**) *Stab2*^*+/+*^ and *Stab2*^*−/−*^ myoblasts were induced to differentiate for the indicated times. The levels of embryonic MyHC (*Myf3*, **d**) and myogenin (*MyoG*, **e**) mRNA were analysed by quantitative real-time PCR. Data are presented as mean±s.d. of three independent experiments. Asterisks indicate statistical significance (*P<0.05, **P<0.01, ***P<0.001, Student's *t*-test).

To determine whether ablation of Stab2 induces impaired myoblast fusion, we examined the ability of myoblasts from *Stab2*^*+/+*^ and *Stab2*^*−/−*^ mice to form myotubes *in vitro*. On day 2 after the induction of differentiation (DM2), *Stab2*^*+/+*^ myoblasts had formed large myotubes, whereas *Stab2*^*−/−*^ myoblasts had produced only small, thin myotubes (Fig. 5a). The fusion index was significantly lower in Stab2-deficient myoblasts (39.4±6.8) compared with *wild-type* myoblasts (52.4±0.84; Fig. 5b). In *Stab2*^*+/+*^ myoblasts, 65% of the nuclei in MyHC-positive myotubes were observed in myotubes with >15 nuclei at DM2, whereas in *Stab2*^*−/−*^ myoblasts, most of the nuclei in MyHC-positive myotubes were observed in cells containing 2–15 nuclei (Fig. 5c), indicating that ablation of Stab2 leads to a decrease in myonuclear accretion during differentiation. Similar results were observed in C2C12 cells subjected to short hairpin RNA (shRNA)-mediated knockdown of Stab2 (Supplementary Fig. 9). Consistent with the observed decrease in myotube formation, eMyHC expression was significantly reduced in *Stab2*^*−/−*^ myoblasts (Fig. 5d). However, as shown in results from Stab2 overexpression, no difference was observed in the mRNA level of myogenin between *Stab2*^*−/−*^ and *Stab2*^*+/+*^ myoblasts (Fig. 5e), indicating that Stab2 expression had no effect on myogenic commitment. Collectively, our results indicate that Stab2 acts as a membrane protein for myoblast fusion.

### Muscle regeneration is impaired in Stab2-deficient mice

**Figure 6:**
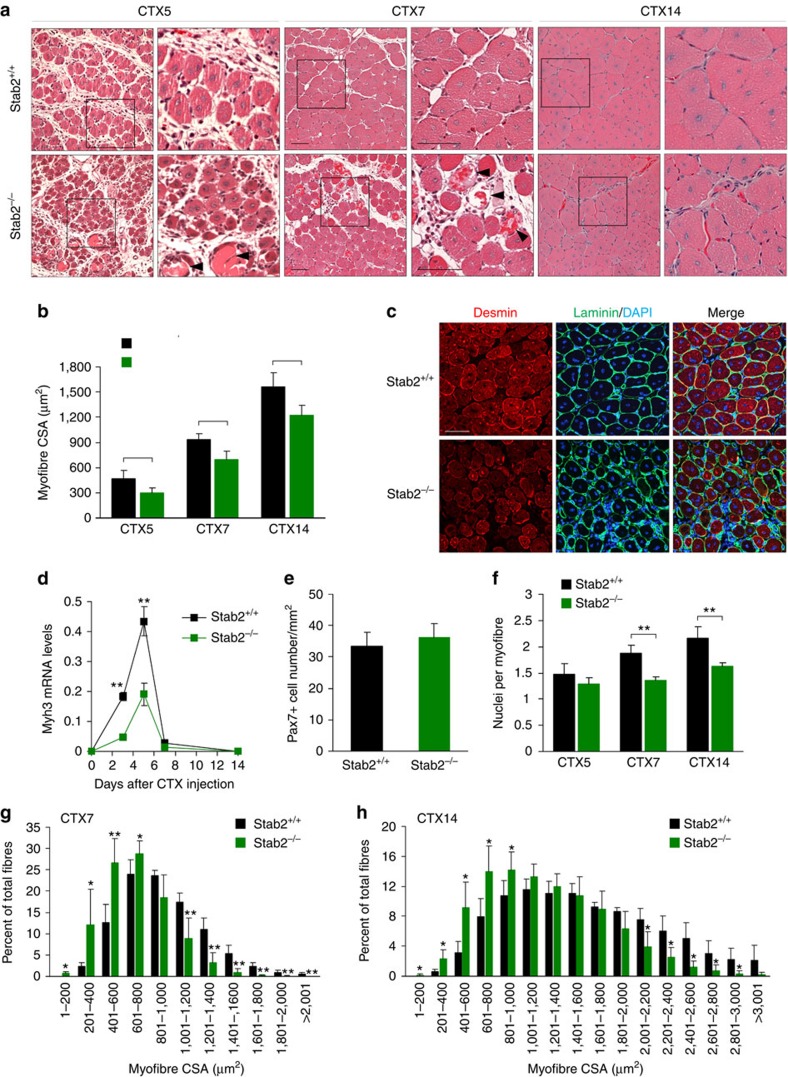
Muscle regeneration is impaired in Stab2^*−/−*^ mice. (**a**) H&E staining of cross sections of *Stab2*^*+/+*^ and *Stab2*^*−/−*^ TA muscles at CTX5, CTX7, and CTX14 (days 5, 7, and 14 after CTX injection). Representative sections are shown. Black boxes are shown at higher magnification. Black arrowheads indicate necrotic myofibres. Scale bars, 50 μm. (**b**) Cross-sectional areas (CSAs) of regenerating myofibres were analysed at CTX5, CTX7, and CTX14 using the Image J program. Data are presented as mean±s.d. (*n*=5 per time point) of each group. (**c**) Immunostaining for desmin and laminin in *Stab2*^*+/+*^ and *Stab2*^*−/−*^ TA muscles at 7 days after CTX injection. Scale bar, 50 μm. (**d**) The levels of embryonic MyHC (*Myh3*) mRNA were analysed in TA muscle of wild-type and Stab2-deficient mice during muscle regeneration after CTX injury. Data are presented as mean±s.d. of at least three independent experiments. (**e**) The number of pax7-positive satellite cells per mm^2^ was counted for TA muscles of wild-type and Stab2-deficient mice (*n*=5). (**f**) DAPI-stained nuclei within dystrophin-positive sarcolemma were analysed in regenerating muscles from *Stab2*^*+/+*^ and *Stab2*^*−/−*^ mice, and numbers of myonuclei per myofibre were calculated. Data are presented as mean±s.d. (*n*=5) of each genotype. (**g**,**h**) The distributions of fibre sizes at CTX7 (**g**) and CTX14 (**h**) were analysed. More than 2,000 fibres were measured in each sample (*n*=5). Data are presented as mean±s.d. (*n*=5). Asterisks indicate statistical significance (*P<0.05, **P<0.01, Student's *t*-test).

Myoblast fusion is also important for the post-injury healing and regeneration of muscle^38^. To examine the function of Stab2 during skeletal muscle regeneration, we injected the tibialis anterior muscles of 9-week-old male *Stab2*^*+/+*^ and *Stab2*^*−/−*^ mice with cardiotoxin (CTX) to induce injury, and analysed muscle regeneration after injury (Supplementary Fig. 10a). In day 3 post injury, the site of injury consisted of a mononuclear infiltrate composed of inflammatory cells and activated myoblasts. Muscle injury was comparable for both *Stab2*^*+/+*^ and *Stab2*^*−/−*^ mice (Supplementary Fig. 10b). Five and seven days following CTX injection, most of the inflammatory cells in *Stab2*^*+/+*^ mice were cleared and the damaged myofibres had been replaced by newly formed myofibres with centralized nuclei (Fig. 6a, top row). In *Stab2*^*−/−*^ mice at days 5 and 7 post injection, in contrast, the newly formed myofibres were smaller, and some inflammatory cells and necrotic myofibres were still present (Fig. 6a, bottom row). The muscle architecture was largely restored in both *Stab2*^*+/+*^ and *Stab2*^*−/−*^ mice on day 14 post injection, but the myofibres in *Stab2*^*−/−*^ mice remained smaller than those in *Stab2*^*+/+*^ mice (Fig. 6a). CSA analyses revealed that myofibre regeneration was impaired in *Stab2*^*−/−*^ mice compared with *Stab2*^*+/+*^ mice (*n*=5 animals, >2,000 fibres scored per sample; Fig. 6b). To gain additional insight into this impairment in the muscle regeneration of *Stab2*^*−/−*^ mice, we assessed the expression of the intermediate filament protein, desmin, which is expressed in newly formed myofibres during myogenesis and muscle regeneration^39^. Desmin was strongly expressed in most regenerating myofibres of *Stab2*^*+/+*^ mice on day 7 post injection, whereas those of *Stab2*^*−/−*^ mice showed relatively lower expression of desmin, and the desmin-positive myofibres were heterogeneous in size (Fig. 6c). Furthermore, eMyHC mRNA was significantly decreased in *Stab2*^*−/−*^ mice compared with wild-type mice at early stage of regeneration (CTX3 and CTX5; Fig. 6d). To investigate whether the pool of satellite cells was affected in the *Stab2*^*−/−*^ mice, we evaluated the number of Pax7-positive cells in uninjured tibialis anterior muscles from 9-week-old male *Stab2*^*+/+*^ and *Stab2*^*−/−*^ mice. No difference was observed in the number of Pax7-positive cells between *Stab2*^*+/+*^ and *Stab2*^*−/−*^ mice (Fig. 6e and Supplementary Fig. 11a). Expression levels of Pax7 mRNA did not significantly differ between *Stab2*^*−/−*^ and *Stab2*^*+/+*^ tibialis anterior muscles during regeneration after CTX injury (Supplementary Fig. 11b). Expression levels of MyoD and myogenin, which are markers of satellite cell activation and differentiation, respectively, were not affected during muscle regeneration by ablation of Stab2 (Supplementary Fig. 11c,d). To determine whether reduced CSA in *Stab2*^*−/−*^ mice is due to decreased myoblast fusion, we analysed the proportion of centrally nucleated myofibres and number of myonuclei in newly formed myofibres. Although some necrotic myofibres were detected in *Stab2*^*−/−*^ mice, most myofibres were centrally nucleated from 5 days after CTX injury in both mice (Supplementary Fig. 10c). However, myonuclei number in newly formed myofibres and percentages of centralized nuclei were significantly decreased in *Stab2*^*−/−*^ muscles at 7 and 14 days post injury (Fig. 6f and Supplementary Fig. 10d), suggesting that impaired regeneration in *Stab2*^*−/−*^ muscles is due to defective myoblast fusion. In agreement with results from myonuclei analysis, our analysis of myofibre distribution revealed that the regenerating muscles of *Stab2*^*−/−*^ mice were characterized by more small myofibres and fewer large myofibres, compared with those of *Stab2*^*+/+*^ mice (*n*=5 animals, >2,000 fibres scored per sample; Fig. 6g,h). Collectively, these data suggest that Stab2 contributes to the proper post injury regeneration of skeletal muscle.

### Stab2 contributes to phosphatidylserine-dependent fusion

**Figure 7:**
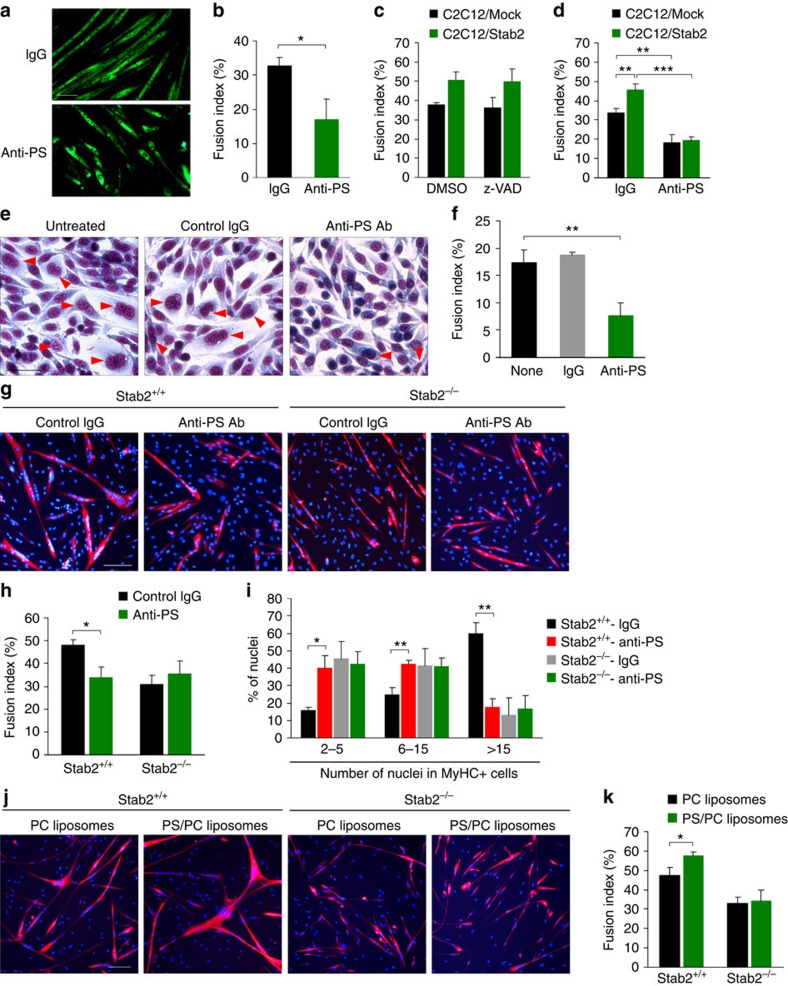
Stabilin-2 contributes to phosphatidylserine-dependent myoblast fusion. (**a**,**b**) C2C12 cells were incubated with differentiation medium for 5 days in the presence of anti-PS antibody or isotype-matched IgG. Representative images (**a**) are shown. Scale bars, 100 μm. Fusion indices (**b**) were calculated. Data are presented as mean±s.d. (*n*=3). (**c**) The fusion indices of C2C12/Mock and C2C12/Stab2 cells in the presence of pan-caspase inhibitor (z-VAD-fmk, 30 μM) or DMSO were calculated at DM5 (*n*=3). (**d**) The fusion indices of C2C12/Mock and C2C12/Stab2 cells in the presence of anti-PS antibody (10 μg ml^−1^) or isotype-matched IgG (10 μg ml^−1^) were calculated at DM5 (*n*=4). (**e**,**f**) L/Stab2 cells were incubated with fusion medium for 48 h in the presence of anti-PS antibody or isotype-matched IgG. Representative images (**e**) are shown. Red arrowheads indicate multinucleated cells. Scale bars, 100 μm. Fusion indices (**f**) were calculated. Data are presented as mean±s.d. (*n*=3). (**g**) *Stab2*^*+/+*^ and *Stab2*^*−/−*^ myoblasts were induced to differentiate in the presence of anti-PS antibody or isotype-matched IgG. After 48 h, cells were stained with anti-MyHC antibody. Representative microscopic fields are shown. Scale bars, 100 μm. (**h**) At DM2, the fusion indices of *Stab2*^*+/+*^ and *Stab2*^*−/−*^ myoblasts in the presence of anti-PS antibody or isotype-matched IgG were calculated. Data are presented as mean±s.d. (*n*=3). (**i**) *Stab2*^*+/+*^ and *Stab2*^*−/−*^ myoblasts were fixed and immunostained for MyHC after 2 days of differentiation (DM2), and the percentage of nuclei present in MyHC-positive cells with the indicated number of nuclei were calculated. Data are presented as mean±s.d. (*n*=3). (**j**) *Stab2*^*+/+*^ and *Stab2*^*−/−*^ myoblasts were induced to differentiate in the presence of PS/PC liposomes (30 μM) or PC liposomes (30 μM). After 48 h, cells were stained with anti-MyHC antibody. Representative microscopic fields are shown. Scale bars, 100 μm. (**k**) At DM2, the fusion indices of *Stab2*^*+/+*^ and *Stab2*^*−/−*^ myoblasts in the presence of in the presence of PS/PC liposomes or PC liposomes were calculated. Data are presented as mean±s.d. (*n*=3). Asterisks indicate statistical significance (*P<0.05, **P<0.01, ***P<0.001, Student's *t*-test).

Although Stab2 is a phosphatidylserine receptor for apoptotic cell clearance, Stab2 can act as scavenger receptor for several ligands, including hyaluronic acid and chondroitin sulfates^40^,^41^. To determine whether Stab2 acts as a phosphatidylserine receptor for myoblast fusion, C2C12 cells were induced to differentiate in the presence of recombinant phosphatidylserine-binding EGF-like domain repeat^30^ or Link domain of Stab2, which is domain for binding of hyaluronic acid and chondroitin sulfates^42^. Myoblast fusion was significantly inhibited by EGF-like domain repeat, but not Link domain (Supplementary Fig. 12), suggesting that an interaction between Stab2 and phosphatidylserine may induce cell fusion. During myogenic differentiation, phosphatidylserine exposure on the cell surface was induced in both healthy and apoptotic myoblasts^6^,^7^,^20^. In agreement with previous findings, we found that phosphatidylserine externalization was induced in non-apoptotic myoblasts as well as dying cells after induction of differentiation (Supplementary Fig. 13). Blockade of phosphatidylserine with an anti-phosphatidylserine antibody significantly inhibited myoblast fusion in C2C12 cells (Fig. 7a,b). Recently, signalling from apoptotic myoblasts induces fusion between healthy myoblasts^20^. To determine the involvement of phosphatidylserine-exposed apoptotic myoblasts in Stab2-mediated myoblast fusion, the effect of pan-caspase inhibitor, z-VAD-fmk, on myoblast fusion in C2C12/Mock and C2C12/Stab2 cells was analysed. Treatment with pan-caspase inhibitor did not inhibit myoblast fusion in both C2C12/Mock and C2C12/Stab2 cells (Fig. 7c and Supplementary Fig. 14a). In contrast, blockade of phosphatidylserine using anti-phosphatidylserine antibody inhibited myoblast fusion in both cells, and fusion index of C2C12/Stab2 cells was reduced to a level similar to that of C2C12/Mock cells (Fig. 7d and Supplementary Fig. 14b). These findings suggested the possibility that Stab2 can induce myoblast fusion via recognition of phosphatidylserine on the cell surface of healthy myoblasts rather than apoptotic myoblasts. Furthermore, we induced cell fusion in L/Stab2 cells in the presence of anti-phosphatidylserine antibody and analysed the formation of multinucleated cells. Treatment of L/Stab2 cells with the phosphatidylserine-blocking antibody decreased cell fusion by 56%, whereas control IgG did not (Fig. 7e,f). Next, to assess whether Stab2 is involved in the phosphatidylserine-dependent fusion of myoblasts, we examined the effect of phosphatidylserine blockade on the fusion of *Stab2*^*+/+*^ and *Stab2*^*−/−*^ myoblasts. Treatment with the phosphatidylserine-blocking antibody decreased the fusion of Stab2^*+/+*^ myoblasts by 26%, but had no effect on *Stab2*^*−/−*^ myoblasts (Fig. 7g,h). When *Stab2*^*+/+*^ myoblasts were treated with the phosphatidylserine-blocking antibody, the percentage of nuclei in large myotubes (>15 nuclei) was reduced to a level similar to that seen of *Stab2*^*−/−*^ myoblasts (Fig. 7i). In contrast, the phosphatidylserine-blocking antibody had no effect on the proportion of nuclei in MyHC-positive myotubes during *Stab2*^*−/−*^ myoblast fusion (Fig. 7i). To examine the effect of exogenous phosphatidylserine on myoblast fusion, we evaluated the fusion of *Stab2*^*+/+*^ and *Stab2*^*−/−*^ myoblasts in the presence of phosphatidylserine/phosphatidylcholine liposomes, using phosphatidylcholine liposomes as a negative control. Treatment with phosphatidylserine/phosphatidylcholine liposomes increased the fusion of *Stab2*^*+/+*^ myoblasts by 21%, but had no effect on *Stab2*^*−/−*^ myoblasts (Fig. 7j,k). Together, these results indicate that Stab2 contributes to the phosphatidylserine-dependent fusion of myoblasts.

## Discussion

Several lines of evidence have indicated that the externalization of phosphatidylserine functions in myoblast fusion, suggesting that phosphatidylserine receptors on myoblasts may be important for this cell–cell fusion. The results of the present study provide evidence that the phosphatidylserine receptor Stab2 contributes to myoblast fusion and skeletal muscle regeneration. We herein show that the muscles of *Stab2*^*−/−*^ mice have myofibres with small CSA and fewer myonuclei, compared with those of *Stab2*^*+/+*^ mice, indicating that the reduced CSA of *Stab2*^*−/−*^ muscle appears to be due to decreased myoblast fusion. In agreement with these findings, forced expression of Stab2 enhanced the formation of large myotubes, and the shRNA-mediated knockdown or ablation of Stab2 in myoblasts resulted in the formation of thin myotubes with fewer nuclei. Furthermore, deficiency of Stab2 resulted in impaired muscle regeneration after injury *in vivo*.

Three phosphatidylserine receptors have been identified as being involved in recognizing phosphatidylserine on the cell surface of apoptotic cells: Tim4 (ref. 11), Bai1 (ref. 12) and Stab2 (ref. 13). Under our experimental condition, the Bai1 mRNA was barely expressed in skeletal muscles and during myogenic differentiation. Consistent with this result, a recent study failed to detect Bai1 transcripts in C2C12 cells and MyoD-positive muscle precusors^43^. We further found that Tim4 was only minimally expressed in skeletal muscles, and its expression decreased after the induction of differentiation. In contrast, Stab2 was highly expressed in adult muscle tissues, and it was upregulated during differentiation, suggesting that Stab2 may act as a membrane protein for phosphatidylserine recognition in myoblast fusion. Consistent with this, three main lines of evidence indicate that Stab2 contributes to phosphatidylserine-dependent fusion in muscle cells. First, forced expression of Stab2 induced the formation of multinucleated cells in fibroblasts, and this fusion was inhibited by an anti-phosphatidylserine-blocking antibody. Consistent with a previous finding that the recognition of phosphatidylserine by EGF-like repeat domains in Stab2 is increased at low pH^37^, the cell–cell fusion in Stab2-expressing fibroblasts was enhanced in a low-pH fusion medium. Second, blockade of phosphatidylserine using an anti-phosphatidylserine antibody significantly inhibited the fusion of wild-type myoblasts, whereas the decreased cell fusion by this phosphatidylserine blockade did not occur in Stab2-deficient myoblasts. Third, treatment with phosphatidylserine/phosphatidylcholine liposomes increased the fusion index in wild-type myoblasts, whereas enhanced fusion by exogenous phosphatidylserine was not observed in Stab2-deficient myoblasts. Thus, our results indicate that Stab2 contributes to phosphatidylserine-dependent fusion during myogenic differentiation.

Recently, the activation of phosphatidylserine receptor Bai1 by apoptotic myoblasts was shown to promote the fusion of healthy myoblast^20^. Both Stab2 and Bai1 act as phosphatidylserine receptors and depend on their ability to bind phosphatidylserine to enhance myoblast fusion. Indeed, phosphatidylserine-binding domain of both receptors (thrombospondin type I repeats in Bai1 (ref. 20) and EGF-like domain repeats in Stab2) inhibited myoblast fusion during myogenic differentiation. However, fusion-promoting effect of Bai1 depends on apoptotic myoblasts during myogenic differentiation, whereas Stab2-mediated fusion is not. Under our experimental conditions, pan-caspase inhibitor did not inhibit myoblast fusion in C2C12/Stab2 and C2C12/Mock cells. This finding indicates that mechanisms regarding phosphatidylserine/Bai1 and phosphatidylserine/Stab2 for myoblast fusion are different. Phosphatidylserine exposure on cell surface is also observed in non-apoptotic myoblasts during myogenic differentiation^6^,^7^. We also found that non-apoptotic myoblasts exposed phosphatidylserine on the cell surface during differentiation, and fusion-promoting effect of Stab2 was abrogated by phosphatidylserine antibody. Although the mechanism of how Stab2 regulates myoblast fusion is incomplete, our findings suggest that Stab2 accelerates myoblast fusion via recognition of phosphatidylserine on healthy myoblasts rather than apoptotic myoblasts. In addition, the fusion phenotype in Stab2-deficient mice is relatively mild in comparison with defects observed in Rac, Cdc42, Myomaker, N-Wasp or Dock1 deficiency, which are all embryonic lethal. Thus, it is possible that Stab2 is not essential for myoblast fusion *in vivo* but is instead important to modulate fusion efficiency via phosphatidylserine recognition. It was known that some scavenger receptors, such as CD36, can also recognize phosphatidylserine during cell–cell fusion^10^,^44^. Alternatively, other receptors may act redundantly with Stab2.

Calcineurin-dependent signalling has been implicated in myoblast differentiation^45^ and skeletal muscle growth and hypertrophy^46^,^47^,^48^. Calcineurin dephosphorylates NFATs and translocate them to the nucleus, thereby allowing the activation of target genes. NFATc1, NFATc2, NFATc3 and NFAT5 are known to be expressed in skeletal muscle^49^,^50^. In this study, we show that NFATc1 regulates the transcriptional activation of the Stab2 gene via a direct interaction with nucleotides −188 to −183 of the Stab2 promoter. Furthermore, knockdown of NFATc1 resulted in decrease in Stab2 expression as well as myoblast fusion during myogenic differentiation. Several studies have indicated that calcineurin/NFATc1 pathway regulates muscle growth. For example, activation of the calcineurin/NFATc1 pathway by insulin-like growth factor-1 was shown to promote skeletal muscle hypertrophy^48^, and Four-and-a-half LIM protein 1 was found to regulate the skeletal muscle mass via the NFATc1 pathway^51^,^52^. In addition, calcineurin also regulates skeletal muscle regeneration via associations with NFATc1 and GATA-2 (ref. 53). In the present study, we show that overexpression of stab2 increased the size of the formed myotubes and the number of myonuclei per myotube. The phenotypes of *Stab2*^*−/−*^ mice further support our contention that Stab2 has a role in myoblast fusion during muscle growth and regeneration *in vitro* and *in vivo*. For example, the CSA of normal or regenerating muscles were reduced in *Stab2*^*−/−*^ mice, and this muscle was characterized by myofibres with fewer myonuclei, suggesting decreased myonuclear accretion in *Stab2*^*−/−*^ muscle. Consistent with this finding, *Stab2*^*−/−*^ myoblasts do not efficiently form large myotubes. Myoblast fusion is important not only for myogenesis during development but also for muscle growth and regeneration. Thus, it is possible that Stab2 is a downstream target of the calcineurin/NFATc1 signalling for the regulation of muscle growth and regeneration.

In summary, we herein show that Stab2 appears to have a role in myoblast fusion during myogenic differentiation and muscle regeneration. Stab2 was found to be expressed via NFATc1 and to contribute to phosphatidylserine-dependent myoblast fusion. Therefore, modulation of Stab2 expression may represent novel therapeutic strategies for regulating skeletal muscle mass in myopathies.

## Methods

### Antibodies

Normal goat IgG and mouse IgG were obtained from Chemicon. Normal rabbit IgG was purchased from DAKO. Polyclonal anti-stabilin-1 (1bR1) and anti-Stab2 (16R-2) antibodies were generated by immunizing rabbit with the peptides CEPFDDSVLEEDFPDT and CDPFTDSGERELENSD, respectively^29^, and affinity-purified in Abfrontier (Seoul, Korea). Anti-myogenin (F5D, sc-12732), anti-YY1 (H-414, sc-1703) and anti-NFATc1 (7A6, sc-7294) antibodies were obtained from Santa Cruz Biotechnology. Monoclonal anti-phosphatidylserine antibody (1H6, 05–719) was purchased from Upstate. Anti-MyHC (MF20), anti-eMyHC (F1.625) and anti-pax7 (PAX7) antibodies were obtained from Developmental Studies Hybridoma Bank. Anti-α-tubulin (DM1A, T6199), anti-laminin (L9393), anti-dystrophin (MANDYS8, D8168), anti-actin (A2066) and anti-FLAG (M2, F1804) antibodies were purchased from Sigma. Anti-desmin (DE-U-10, ab6322) and polyclonal anti-Tim-4 (ab47637) antibodies were purchased from Abcam. Polyclonal anti-Bai1 antibody (NBP1–00723) was obtained from Novus Biologicals. Alexa Fluor 488-, Alexa Fluor 568-, Alexa Fluor 594- and Alexa Fluor 647-conjugated secondary antibodies were obtained from Invitrogen.

### Cell culture and transfection

C2C12 myocytes were purchased from ATCC (CRL-1772) and maintained in DMEM medium containing 10% (v/v) FBS and antibiotics (100 units per ml of penicillin and 100 μg ml^−1^ of streptomycin). To induce myogenic differentiation, cells were grown to 90% confluence in maintenance medium and then switched to differentiation medium (DMEM supplemented with 2% (v/v) horse serum). Human myoblasts were purchased from Clontech and induced to differentiate according to manufacturer's instruction. 293FT cells were purchased from Invitrogen and maintained in DMEM medium containing 0.1 mM MEM non-essential amino acids, 10% FBS and antibiotics. Plat-E cells were maintained in DMEM medium containing 10% FBS, 1 μg ml^−1^ puromycin, and 10 μg ml^−1^ blasticidin. Mouse fibroblast L cells expressing Stab2 (L/Stab2 cells)^13^ were maintained in DMEM medium containing 10% (v/v) FBS and antibiotics. To induce cell fusion, cells were grown in fusion medium (DMEM supplemented with 2% (v/v) horse serum, pH 6.8). To generate Stab2-overexpressing C2C12 cells (C2C12/Stab2), plasmid encoding human Stab2-FLAG was transfected into C2C12 cells using Lipofectamine 2000, in accordance with the manufacturer's instructions (Invitrogen). Cells were selected in G418 (800 μg ml^−1^) for 2 weeks and used further experiments. Stab2 expression was assessed by western blotting using anti-FLAG antibody (1:10,000, Sigma) and qrtPCR.

### Isolation of primary myoblasts

Primary myoblasts were derived from the hindlimb muscles of newborn *Stab2*^*+/+*^ or *Stab2*^*−/−*^ mice. In brief, muscles were minced mechanically and digested with enzyme mixture (1.5 U ml^−1^ collagenase D, 2.4 U ml^−1^ dispase II and 2.5 mM CaCl_2_) in Ham's F10 nutrient mixture for 45 min at 37 °C with slight agitation. Muscles were further dissociated by trituration and passed through a 70 μm mesh. Cells were suspended in growth media (Ham's F10 supplemented with 20% FBS and 5 ng ml^−1^ basic fibroblast growth factor) and grown on collagen-coated dishes (IWAKI) in a humidified 5% CO_2_ incubator at 37 °C. To induce myogenic differentiation, cells were plated on dishes coated with entactin–collagen IV–laminin matrix (Upstate Biotechnology) in growth medium and shortly thereafter switched to DM (DMEM supplemented with 1% insulin–transferrin–selenium-A (Invitrogen)).

### Quantitative real-time PCR

Total RNA was purified from C2C12 cells, primary myoblasts and skeletal muscle tissues using Trizol reagent, in accordance with the manufacturer's instructions (Invitrogen). Reverse transcription was performed with M-MLV reverse transcriptase (Promega) using 2 μg of total RNA (Invitrogen) for 50 min at 42 °C, followed by 3 min at 95 °C. Complementary DNA (cDNA) produced by reverse transcription was diluted fivefold, and SYBR green master mix (Roche Applied Science) was then used to amplify stabilin-1, Stab2, Tim1, Tim4, Bai1, Myh3, MyoG, MyoD, Pax7 or GAPDH (the internal control). Real-time PCR amplification was performed in a LightCycler 480 (Roche Applied Science) using the following conditions: initial denaturation at 95 °C for 5 min; 45 cycles of amplification with denaturation at 95 °C for 30 s, annealing at 58 °C for 30 s and extension at 72 °C for 30 s; 1 cycle of melting curves at 95 °C for 5 s, 65 °C for 1 min and 97 °C continuous; and a final cooling step at 40 °C for 30 s. qrtPCR results were analysed using the comparative cycle threshold (*C*_T_) method. Briefly, the expression of a target gene relative to that of the reference gene (GAPDH) was calculated using the 2^−ΔCT^ formula (where ΔCT*=*ΔCT_target_—ΔCT_ref_). The primers used for rtPCR are described in Supplementary Table 1.

### Western blot

C2C12 cells or myoblasts were lysed at 4 °C in cold lysis buffer containing 50 mM Tris-HCl (pH 7.4), 150 mM NaCl, 1% Triton X-100, 1 mM phenylmethylsulphonyl fluoride and proteinase inhibitor cocktails (Roche). Identical amounts of total cell lysates were resolved by 6% or 10% SDS–PAGE and then transferred onto nitrocellulose membranes, which were then incubated in blocking solution consisting of 5% skim milk in TBS-T (10 mM Tris-HCl (pH 8.0), 150 mM NaCl, and 0.1% Tween 20) for 1 h at room temperature, and immunoblotted using anti-stabilin-1 antibody (1:1,000), anti-Stab2 antibody (1:1,000), anti-MyHC antibody (1:10,000), anti-myogenin antibody (1:1,000), anti-α-tubulin antibody (1:5,000), anti-NFATc1 (1:1,000), anti-YY1 (1:1,000), anti-Tim4 (1:500), anti-Bai1 (1:500) or anti-actin antibody (1:5,000). Immunoreactive signals were visualized using an enhanced chemiluminescence detection reagent (Amersham Pharmacia Biotech).

### Immunofluorescent staining

C2C12 cells or primary myoblasts were incubated in differentiation medium to induce muscle cell fusion for the indicated times. Cell were fixed in 4% formaldehyde in PBS for 10 min at room temperature, and permeabilized with 0.1% Triton X-100. Non-specific binding was minimized by incubating the cells in PBS containing 2% BSA for 1 h. After three washes with PBS, cells were incubated overnight at 4 °C with a polyclonal anti-Stab2 antibody (1:100) and a monoclonal anti-MyHC antibody (1:100), and then washed with PBS (3 × 10 min) at room temperature. Cells were incubated with Alexa Fluor 568-conjugated anti-rabbit IgG (1:200) and Alexa Fluor 488-conjugated anti-mouse IgG (1:200) for 1 h at room temperature, washed with PBS (3 × 10 min) and treated with a solution of SlowFade (Molecular Probes). Cells were viewed under a fluorescence microscope (Leica).

### Fusion assays

C2C12 cells or primary myoblasts were incubated in differentiation medium to induce muscle cell fusion for the indicated times, fixed in 4% formaldehyde in PBS for 10 min at room temperature, and immunostained with anti-MyHC antibody (1:100, Developmental Studies Hybridoma Bank). The fusion indices were determined by dividing the number of nuclei in MyHC-positive myotubes with ≥2 nuclei by the total number of nuclei analysed (*n*>500). For nuclear number assays, myoblast fusion was quantified by calculating the percentage of nuclei present in MyHC-positive myotubes with the indicated number of nuclei. L/Mock or L/Stab2 cells were incubated in fusion medium for 48 h and, fusion indices were calculated as described above. In some experiments, to block phosphatidylserine on cell surfaces, myogenic differentiation and cell fusion were induced in the presence of monoclonal anti-phosphatidylserine antibody (10 μg ml^−1^, Upstate) or control mouse IgG (10 μg ml^−1^, Chemicon). For blocking of apoptosis, C2C12 cells were induced to differentiate in the presence with z-VAD-fmk (30 μM, Enzo Life Science) or dimethylsulphoxide. For treatment of exogenous phosphatidylserine, phosphatidylserine/phosphatidylcholine liposomes containing phosphatidylserine and phosphatidylcholine at a molar ratio of 50:50 were prepared. Phosphatidylcholine liposomes (PC 100%) were used as a control. The lipids were mixed in chloroform and then dried using centrifugal vacuum concentrator. Next, the dried lipids were resuspended in PBS at a final concentration of 1 mM, followed by sonication for 10 min on ice. Primary myoblasts were induced to differentiate for 16 h and incubated with differentiation medium in the presence of phosphatidylserine/phosphatidylcholine liposomes (30 μM) or phosphatidylcholine liposomes (30 μM) for another 32 h.

### Stab2 knockdown in C2C12 cells

For knockdown of Stab2, the target sequence of the shRNA for mouse Stab2 was 5′-TGGCAAGGACAGCTGACTTC-3′. Scrambled sequence (5′-TACGAGCGAGTACTACCGGT-3′) was used as a control. Sense and antisense oligonucleotides were annealed, cloned into the pSuper/neo shRNA vector (Oligoengine), and then designated as pSuper/shmStab2 and pSuper/shCont, respectively. C2C12 cells were transfected with pSuper/shmStab2 or pSuper/shCont vector using Lipofectamine 2000 (Invitrogen). For stable transfection, cells were selected in G418 (800 μg ml^−1^). Individual G418-resistant colonies were isolated after 10–12 days of culture. The final clones were designated C2C12/shmStab2 or C2C12/shCont. The repression of Stab2 expression was assessed by western blotting.

### Generation of Fc fusion protein in mammalian cells

The cDNA encoding N-terminal region of Stab2 (amino acids 1–63) were amplified from pcDNA-Stab2 vector, cloned into pcDNA-Fc^30^ and then designated as pcDNA-S-Fc. To generate expression vectors for Fc fusion EGFrp and Link protein, the fragment of Stab2 cDNA encoding amino acids 1,303–1,596 and 2,196–2,291 were generated by PCR, cloned into the *Bam*HI and *Xho*I sites of pcDNA–S–Fc vector and designated as pcDNA–EGFrp–Fc and pcDNA–Link–Fc, respectively. Primer sequences are described in Supplementary Table 1. HEK293 cells were then transfected with pcDNA–EGFrp–Fc or pcDNA–Link–Fc vector for 6 h using Lipofectamine 2000 (Invitrogen), in accordance with the manufacturer's instructions. Twenty-four hours after transfection, culture medium was exchanged with serum-free DMEM. The supernatant was collected after 48 h, and Fc fusion proteins were purified using protein A-conjugated agarose (Amersham Pharmacia) in accordance with the manufacturer's instructions. C2C12 cells were induced to differentiate in the presence of EGFrp-Fc (30 μg ml^−1^) or Link-Fc protein (30 μg ml^−1^).

### Staining of phosphatidylserine-exposed myoblasts or dying myoblasts

C2C12 cells were grown in growth medium or induced to differentiate for the indicated times. Cells were incubated with Fixable Viability Dye eFluor 660 (1:1,000 dilution in PBS; eBioscience) for 30 min at 4 °C and then washed with PBS at room temperature. Cells were then incubated with Alexa Fluor 488-conjugated Annexin V (1:20 dilution in Annexin-binding buffer (10 mM HEPES, 140 mM NaCl, and 2.5 mM CaCl_2_, pH 7.4); Molecular Probe) for 15 min at room temperature. After three-time washes with Annexin-binding buffer, cell were fixed in 4% formaldehyde in PBS for 10 min at room temperature, stained with DAPI and treated with antifade solution. Cells were viewed under a fluorescence microscope (Leica).

### Plasmids

Plasmid encoding NFATc2 was kindly provided by J.Y. Cho (Seoul National University), and plasmids encoding NFATc1 and NFATc3 were purchased from Origene. Plasmid encoding NFAT5 was obtained from the Korean Human Gene Bank, and plasmids encoding activated calcineurin and green fluorescent protein-fusion VIVIT were from Addgene. The transcription start site of the human Stab2 gene was determined by 5′ RACE PCR using cDNA from human spleen, in accordance with the manufacturer's instructions (Invitrogen). On the basis of the 5′ RACE PCR result (Supplementary Fig. 1), the human Stab2 promoter region from −1,342 to 205 bp was amplified from human genomic DNA by PCR. The sequences of the primers used are provided in Supplementary Table 1. The PCR product was cloned into pGL3/basic vector (Promega) via *Asp*718I and *Xho*I sites. The 5′ deletion constructs of Stab2 promoter were constructed by PCR using the primer detailed in Supplementary Table 1. PCR products were cloned into pGL3/basic vector via *Asp*718I-*Xho*I or *Nhe*I–*Xho*I sites. Mutations of the putative NFAT-binding site were carried out by two-step PCR mutagenesis using the primers detailed in Supplementary Table 1. All plasmid constructs were verified by DNA sequencing (Bionics, Korea).

### Reporter gene assays

Primary myoblasts were plated in collagen-coated 24-well plate (IWAKI) at a density of 2 × 10^4^ cells per well, and C2C12 cells were plated in 12-well plates at a density of 5 × 10^4^ cells per well. The next day, cells were transfected with pStab2-Luc vector and NFAT expression vector using Lipofectamine 2000 (Invitrogen). Forty-eight hours after transfection, cells were washed with PBS and lysed in 200 μl of passive lysis buffer (Promega). Luciferase activities were measured using a Dual Luciferase assay kit (Promega). Transfection efficiencies were determined by cotransfecting 0.1 μg of plasmid encoding the Renella luciferase gene (pRL-SV40), and the firefly luciferase activity of each sample was normalized to the Renilla luciferase activity.

### ChIP assays

ChIP assays were performed using the Upstate Biotechnology ChIP assay kit. Briefly, human myoblasts were induced to differentiate for 24 h and cross-linked in culture media with formaldehyde (a final concentration of 1%) at room temperature for 10 min. Cells were then washed with PBS and sonicated to yield DNA fragments ranging in size from 200 to 1,000 base pairs. Cell lysate supernatants were diluted 10-fold in ChIP dilution buffer and precleared by incubation with 75 μl of salmon sperm DNA-protein G-agarose for 30 min at 4 °C with agitation to reduce non-specific interactions between chromatin DNA and the agarose beads. Supernatants were immunoprecipitated with anti-NFATc1 antibody (anti-myogenin antibody was used as an isotype-matched control antibody). Immunoprecipitated complexes were eluted from protein G-agarose beads using elution buffer (1% SDS, 0.1 M NaHCO_3_). Chromatin DNA was separated from protein by phenol/EtOH precipitation and then used as a template for PCR using primers specific for Stab2 promoter. GAPDH primers were used as negative controls. Primer sequences are described in Supplementary Table 1.

### Retroviral infection

For generation of retroviral vector encoding NFATc1, the cDNA encoding human NFATc1 was amplified from pCMV-NFATc1 by PCR. The PCR product was cloned into *Bam*HI and *Xho*I sites of pMXs–IRES–puro (Cell Bio Labs). Primer sequences are described in Supplementary Table 1. For generation of retroviral vector for NFATc1 knockdown, the target sequences of the shRNA for mouse NFATc1 was 5′-CACCAAAGTCCTGGAGATC-3′. Sense and antisense oligonucleotides were synthesized, annealed and cloned into the pSuper/puro shRNA vector (Oligoengine). The expression cassette of pSuper/shNFATc1 was subcloned into *Bam*HI and *Not*I sites of pMxs–U6–puro retroviral vector (Cell Bio Lab). Retrovirus particles were generated by transfection of Plat-E cells (Cell Bio Lab) with retroviral vector encoding shNFATc1, in accordance with the manufacturer's instructions. Primary myoblasts were infected with retrovirus encoding the shRNA against mouse NFATc1 in the presence of polybrene (6 μg ml^−1^). After infection for 24 h, cells were incubated in growth medium for 8 h and induced to differentiate for the indicated time. For generation of C2C12 cell line expressing the shRNA against mouse NFATc1, C2C12 cells were infected with retrovirus encoding the shRNA against NFATc1 for 24 h in the presence of polybrene (6 μg ml^−1^). After infection, the cells were cultured in the presence of puromycin (5 μg ml^−1^). Puromycin-resistant cells were selected for 2 weeks and used for further study. The knockdown of NFATc1 was verified by western blotting.

### Generation of Stab2 knockout mice

Conditional *Stab2* floxed mice were generated by homologous recombination of embryonic stem cells with a C57BL/6 background in Ozgene (Perth, Australia). The targeting construct, which contained two *lox*P sites flanking exon 2 of mouse *Stab2*, was cloned into a vector containing a PGK-neomycin (neo) resistant cassette flanked by Flp recombinase target (FRT) sequences. Embryonic stem cells transfected with the targeting construct were then microinjected into C57BL/6 blastocysts to obtain chimeras that were bred with C57BL/6 mice to generate a germline transmitting *Stab2 floxed/neo* mice. The FRT-flanked PGK-neo resistant cassette was removed by Flp-mediated recombination with *ACTFlpe* transgenic mice^54^. To generate mice carrying deleted *Stab2* exon 2, conditional *Stab2* floxed mice were crossed with *Protamine I-Cre* (*PrmI-Cre*) transgenic mice^55^,^56^, which resulted in the generation of *Stab2* heterozygous mice. Finally, *Stab2* knockout mice were prepared by crossing *Stab2* heterozygous mice. All procedures involving animals were conducted with the approval of Kyungpook National University (KNU 2010-68, KNU 2011-23, KNU 2014-34, and KNU 2014-72) and Dongguk University Gyeongju Campus (IACUC-2012-005).

### Muscle mass and myofibre analysis

The tibialis anterior muscles of 9-week-old male *Stab2*^*+/+*^ and *Stab2*^*−/−*^ mice were harvested tendon-to-tendon and weighed. Muscles were preserved in 4% formaldehyde, bisected at the mid-belly and embedded in paraffin. 5-μm sections from muscle centres were stained with hematoxylin and eosin. The mean CSA of each fibres (*n*>1,000) in five fields from each animal (*n*=5) was determined using the Image J program.

To analyse numbers of myonuclei in tibialis anterior muscles from 9-week-old male *Stab2*^*+/+*^ and *Stab2*^*−/−*^ mice, 5-μm sections from muscle centres were incubated with anti-dystrophin antibody (1:400) at 4 °C overnight. After three washes in TNT buffer (0.1 M Tris-HCl (pH 7.5), 0.15 M NaCl, and 0.05% Tween 20), sections were incubated with Alexa Fluor 594-conjugated anti-mouse IgG (1:200) for 1 h at room temperature, stained with DAPI (Sigma), washed with TNT buffer (3 × 5 min), and then treated with Prolong gold antifade reagent (Invitrogen). The number of DAPI-positive nuclei within the dystrophin-positive sarcolemma was counted for 100–150 myofibres.

### CTX injury and histological analysis

Nine-week-old male *Stab2*^*+/+*^ and *Stab2*^*−/−*^ mice were anaesthetized with Gerolan, and 100 μl of 10 μM CTX (CalBiochem) in PBS was injected into right tibialis anterior muscles. Animals were sacrificed by cervical dislocation at 5, 7, and 14 days post-injection. Tibialis anterior muscles were harvested tendon-to-tendon, fixed in 4% formaldehyde and routinely processed for paraffin embedding. 5-μm sections from muscle centres were stained with hematoxylin and eosin, and the CSA of myofibres (*n*>2,000) in tibialis anterior muscles from five animals per experimental condition were determined using the Image J program. For desmin and laminin staining, antigen retrieval was performed by heating slides in TEG buffer (10 mM Tris, 5 mM EGTA, pH 9.0), and non-specific immunoglobulin binding was prevented by blocking with PBS supplemented with 5% normal goat serum. Sections were incubated for 1 h at room temperature with anti-laminin antibody (1:50) and anti-desmin antibody (1:50), and then washed with PBS (3 × 10 min) at room temperature. Sections were incubated with Flour Alexa 488- and 594-conjugated secondary antibody (1:200), and immunofluorescent staining was visualized under a Zeiss confocal microscope.

### Promoter enzyme immunoassays

To produce biotin-labelled NFAT probes, sense and antisense oligonucleotides containing wild-type or mutant NFAT-binding sites were synthesized and annealed in annealing buffer (100 mM NaCl, 1 mM EDTA, 10 mM Tris-HCl, pH 8.0). The sequences of the oligonucleotides are provided in Supplementary Table 1. Nuclear extracts were isolated from 293FT cells transfected with NFATc1 expression vector. Streptavidin-coated 96-well plates (Thermo Fisher Scientific) were washed three times with PBS-T buffer (PBS and 0.05% Tween 20) and then incubated with biotin-labelled oligonucleotide probes containing wild-type or mutated NFAT-binding site (5 pmol per well) for 1 h at room temperature. After three washes with PBS-T buffer, nuclear extract (20 μg) and poly-deoxyinosinic-deoxycytidylic acid (Sigma) were added, and plates were incubated at 4 °C for 2 h. The plates were then washed with HKMG buffer (10 mM HEPES (pH 7.9), 100 mM KCl, 5 mM MgCl_2_, 10% glycerol, 1 mM DTT, 0.5% Nonidet P-40), incubated with anti-NFATc1 antibody (1:1,000) at 4 °C for 2 h, and then incubated with horseradish peroxidase-conjugated secondary antibody (1:2,000). After three washes with HKMG buffer, plates were incubated with a substrate reagent (R&D Systems) for 1 h at 4 °C. The colorimetric reaction was quenched by adding 2 M H_2_SO_4_. Absorbance at 450 nm was measured using a microplate reader (Bio-Rad).

### Electrophoretic mobility shift assay

EMSA was performed using a LightShift chemiluminescent EMSA kit (Pierce). Nuclear extracts were prepared from 293FT cells transfected with NFATc1 expression vector. Biotin-labelled sense and antisense oligonucleotides were annealed in annealing buffer (100 mM NaCl, 1 mM EDTA, 10 mM Tris-HCl, pH 8.0). The sequences of oligonucleotides are described in Supplementary Table 1. Biotin-labeled probe (20 fmol) was incubated with 6 μg of nuclear proteins for 20 min at room temperature in binding buffer (10 mM Tris (pH 7.4), 50 mM KCl, 1 mM dithiothreitol, 0.05 mg ml^−1^ poly(dI-dC), 5 mM MgCl_2_, and 2.5% glycerol and 0.05% NP40). For competition experiments, 50-, 100- and 200-fold excesses of unlabelled NFAT or non-specific probe were preincubated with nuclear extract for 20 min before adding the biotin-labelled NFAT probe. Reaction mixtures were resolved by 6% polyacrylamide gel pre-electrophoresed for 30 min in 0.5 × Tris borate/EDTA, and electrophoresed at 100 V before being transferred to positively charged nylon membranes (Pierce). Transferred DNA molecules were cross-linked to the membrane at 120 mJ cm^−2^ and detected using horseradish peroxidase-conjugated streptavidin (Pierce).

### Real-time imaging

Cells were placed in an incubation chamber equipped with a time-lapse imaging system (BioStation IM; Nikon, Tokyo, Japan). Images were obtained using a Nikon Ti-E inverted microscope equipped with a CoolSnap HQ camera (Roper Scientific, Trenton, NJ). Images were acquired at 5 min intervals and processed using MetaMorph software (Universal Imaging, Downingtown, PA). Movies were exported and converted into AVI format at 1 frame per 0.16 s.

### Statistical analysis

Statistical significance was assessed using the Student *t*-test, *P* values of<0.05 were considered to be statistically significant.

## Additional information

**How to cite this article:** Park, S.-Y. *et al.* Stabilin-2 modulates the efficiency of myoblast fusion during myogenic differentiation and muscle regeneration. *Nat. Commun.* 7:10871 doi: 10.1038/ncomms10871 (2016).

## Supplementary Material

Supplementary Information Supplementary Figures 1-15 and Supplementary Table.

Supplementary Movie The movie shows cell-cell fusion of stabilin-2-expressing cells. Frames were
