# Supplementary material for: Stabilin-2 modulates the efficiency of myoblast fusion during myogenic differentiation and muscle regeneration
Source: Nat Commun. 2016 Mar 14;7:10871. doi: 10.1038/ncomms10871 (PMC4793076; doi:10.1038/ncomms10871)
Supplement: Supplementary Information — Supplementary Figures 1-15 and Supplementary Table. [file ncomms10871-s1.pdf]

**Supplementary Figure 1**

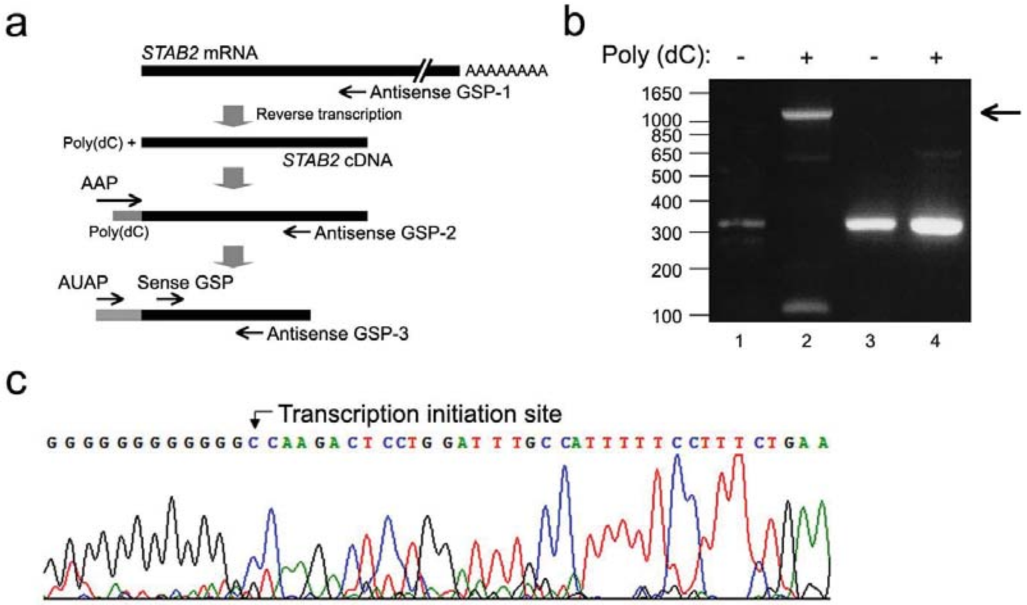


**Supplementary Fig. 1. Identification of the transcription start site in the human *Stabilin-2* gene.** (**a**) The strategy used for 5'-RACE PCR amplification is shown. The locations of the gene-specific primers (GSP) used for 5'-RACE PCR are indicated by arrows. (**b**) The 5'-RACE PCR product was resolved on 2% agarose gel as a specific band (lane 2). Lane 1, 2: AUAP and antisense GSP-3; Lane 3,4: sense GSP and antisense GSP-3. (**c**) The transcriptional start site was identified by sequencing the 5'-RACE PCR product.

**Supplementary Figure 2**

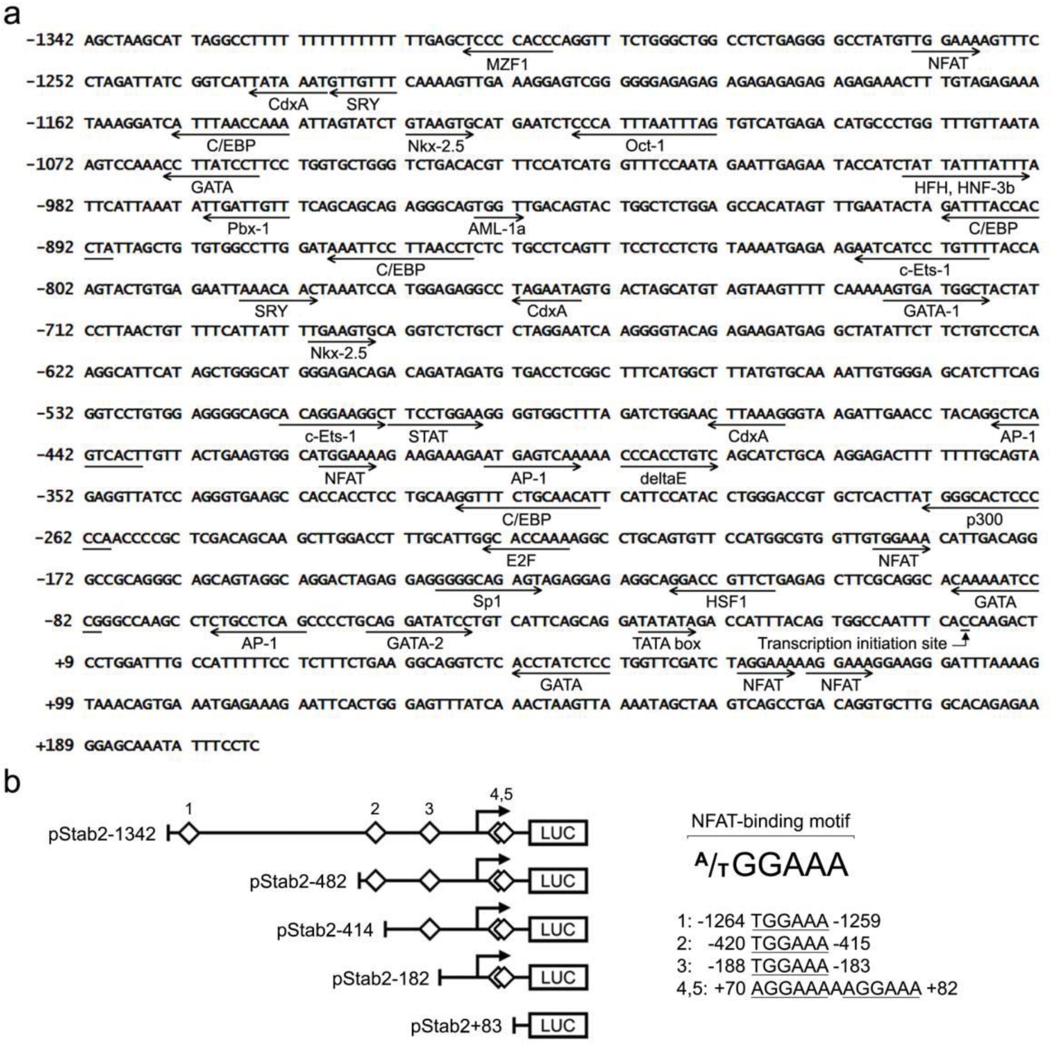


**Supplementary Fig. 2. Characterization of the human stabilin-2 promoter.** (**a**) The human stabilin-2 promoter region. Numbers indicate positions from the transcriptional start site. Putative transcription factor binding sites, which are underlined, were identified using the TFSEARCH program. (**b**) Schematic representations of the human stabilin-2 promoter construct (nt −1342 to +205) and its deleted constructs. Numbers indicate putative NFAT-responsive elements, which are represented by diamonds.

**Supplementary Figure 3**

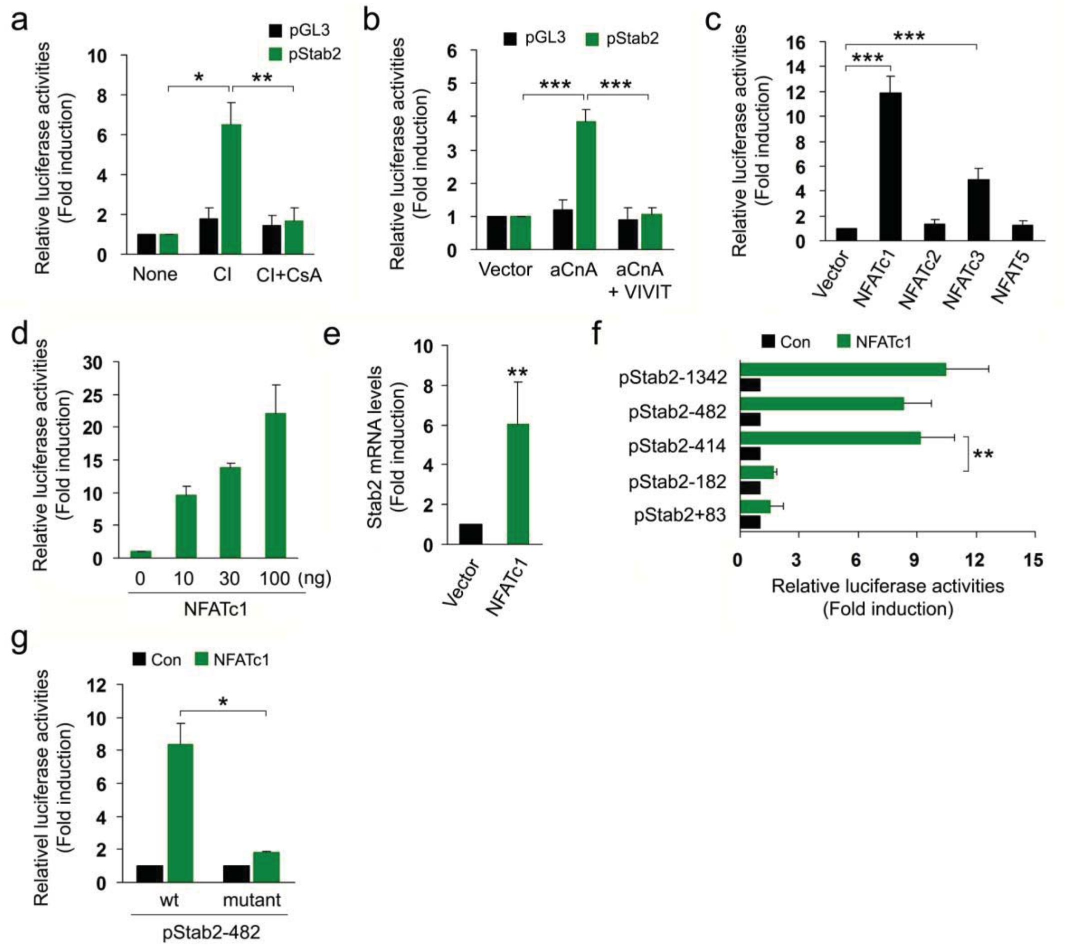


**Supplementary Fig. 3. NFATc1 regulates stabilin-2 expression in C2C12 cells.** (**a**) C2C12 cells were transfected with the stabilin-2 promoter construct. At 24 h post-transfection, cells were incubated with vehicle, calcium ionophore A23187 (CI, 1 μM), or CI plus cyclosporine A (CsA, 1 μM) for 24 h. Relative luciferase activities were normalized as fold over that of vehicle-treated cells. Data are presented as mean ± s.d. (n=3). Asterisks indicate statistical significance (*$P$ < 0.05, **$P$ < 0.01, Student's *t*-test). (**b**) The stabilin-2 promoter construct (nt −1342 to +205) was co-transfected with plasmid encoding activated calcineurin (aCnA) and/or GFP-VIVIT into C2C12 cells. Relative luciferase activities were normalized as fold over that of the control vector. Data are presented as mean ± s.d. (n=4). Asterisks indicate statistical significance (***$P$ < 0.001, Student's *t*-test). (**c**) C2C12 cells were transfected with stabilin-2 promoter construct and the indicated NFAT expression vector. Relative luciferase activities were normalized as fold over that of pcDNA3.1 vector. Data are presented as mean ± s.d.

(n=4). Asterisks indicate statistical significance (***$P < 0.001$, Student's *t*-test). (**d**) The stabilin-2 promoter construct was cotransfected with the indicated amount of plasmid encoding NFATc1 into C2C12 cells. Relative luciferase activities were normalized as fold over that of the stabilin-2 promoter in the absence of NFATc1. Data are presented as mean ± s.d. (n=3). (**e**) C2C12 cells were transfected with plasmid encoding NFATc1 or empty vector. At 48 h post-transfection, total RNAs were isolated, and levels of stabilin-2 mRNA were assessed by quantitative real-time PCR. Expression levels were normalized as fold over that of the cells transfected with pcDNA3.1 vector. Data are presented as mean ± s.d. (n=4). Asterisks indicate statistical significance (**$P < 0.01$, Student's *t*-test). (**f**) The stabilin-2 promoter construct (nt −1342 to +205) and a series of 5' deletion constructs were co-transfected with plasmid encoding NFATc1 into C2C12 cells. Relative luciferase activities were expressed as fold over that of the stabilin-2 promoter in the presence of pcDNA3.1 vector (Con). Data are presented as mean ± s.d. (n=3). Asterisks indicate statistical significance (**$P < 0.01$, Student's *t*-test). (**g**) The stabilin-2 promoter construct (nt −482 to +205) as well as its NFAT mutant were co-transfected with plasmid encoding NFATc1 into C2C12 cells. Relative luciferase activities were normalized as fold over that of each promoter in the absence of NFATc1. Data are presented as mean ± s.d. (n=3). Asterisks indicate statistical significance (*$P < 0.05$, Student's *t*-test).

**Supplementary Figure 4**

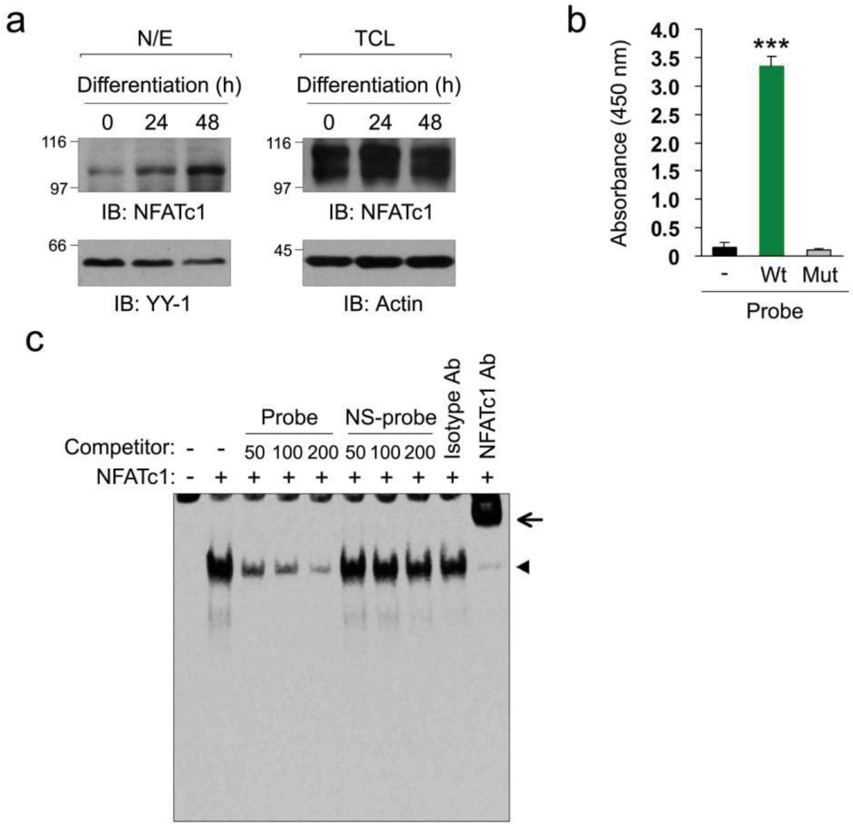


**Supplementary Fig. 4. Characterization of the DNA-binding activities of NFATc1 in stabilin-2 promoter.** (**a**) Primary myoblasts were differentiated for the indicated time, and the amounts of NFATc1 protein in total cell lysates (TCL, right panels) and nuclear extracts (N/E, left panels) were analyzed by Western blotting using an anti-NFATc1 antibody. (**b**) Oligonucleotides containing wild-type (Wt) or mutated (Mut) NFAT-binding site were conjugated onto streptavidin-coated plates, and nuclear extracts from 293FT cells transfected with plasmid encoding NFATc1 were added. After incubation for 2 h, binding between NFATc1 and the oligonucleotide probe was analysed colorimetrically using anti-NFATc1 antibody. Data are presented as mean ± s.d. (n=3). Asterisks indicate statistical significance (***$P < 0.001$, Student's *t*-test). (**c**) EMSAs were performed with biotin-labelled NFAT-responsive element oligonucleotide in 293FT cells transfected with plasmid encoding NFATc1. For competition experiments, unlabelled oligonucleotides were used at 50-, 100-, or 200-fold molar excesses with respect to the labelled oligonucleotide probe. Closed arrowhead indicates the specific DNA/protein complexes. Open arrow indicates the supershifted complexes.

**Supplementary Figure 5**

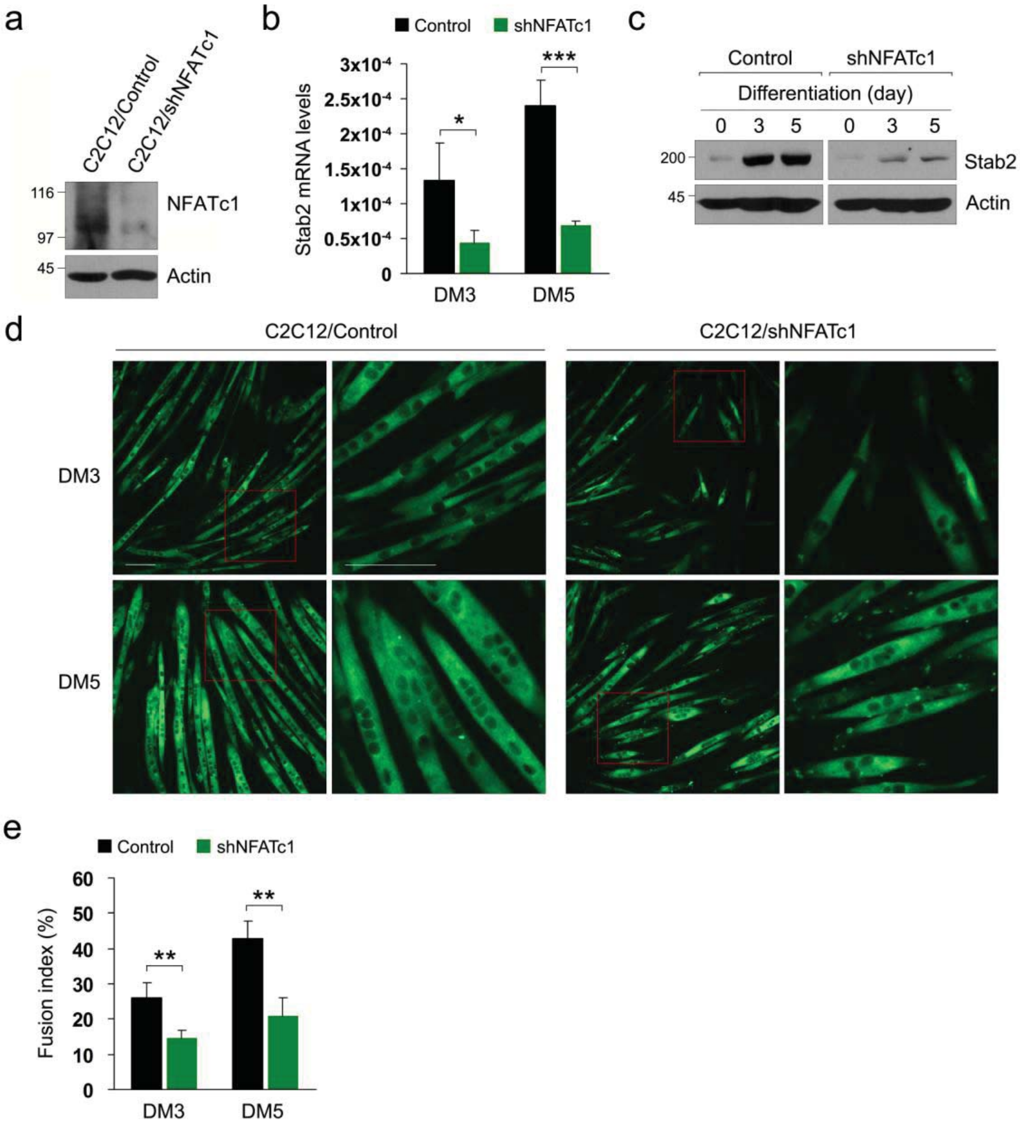


**Supplementary Fig. 5. NFATc1 knockdown inhibits myoblast fusion during myogenic differentiation.** (**a**) C2C12 cells infected with retrovirus encoding shRNA against mouse NFATc1 (C2C12/shNFATc1) or retrovirus from pMXs-U6-Puro vector (C2C12/Control) were generated. NFATc1 expression was evaluated by immunoblotting with anti-NFATc1 antibody. A representative result of three independent experiments is shown. (**b,c**) C2C12/shNFATc1 and C2C12/Control cells were induced to differentiate for the indicated times. Expression of stabilin-2 mRNA (b) and protein (c) were analysed by quantitative real-time PCR and Western blotting, respectively. Data (b) are presented as mean ± s.d. (n=3). Asterisks indicate statistical significance (*$P < 0.05$,

***$P$ < 0.001, Student's *t*-test). (**d**) C2C12/Control and C2C12/shNFATc1 cells were induced to differentiate for the indicated times. Cells were then fixed and immunostained with anti-MyHC antibody. Representative microscopic fields are shown. Scale bar, 100 μm. Red boxes are shown at higher magnification. (**e**) C2C12/shNFATc1 and C2C12/Control cells were induced to differentiate for 3 and 5 days, and fusion indices were calculated. Data are presented as mean ± s.d. (n=3). Asterisks indicate statistical significance (**$P$ < 0.01, Student's *t*-test).

**Supplementary Figure 6**

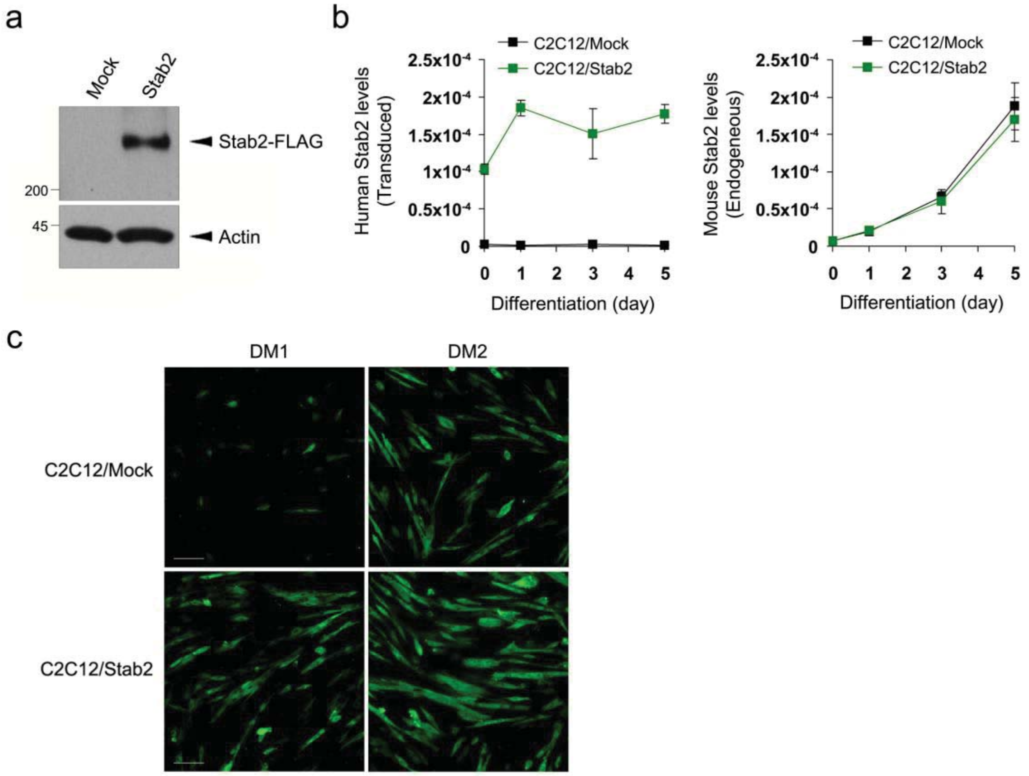


**Supplementary Fig. 6. Overexpression of stabilin-2 in C2C12 cells.** (**a**) C2C12 cells stably transfected with plasmid encoding human stabilin-2-FLAG (C2C12/Stab2) or an empty vector (C2C12/Mock) were generated. Stabilin-2 expression was evaluated by immunoblotting with anti-FLAG antibody (1:10,000). A representative result of three independent experiments is shown. (**b**) Expression levels of transduced human stabilin-2 and endogenous mouse stabilin-2 were determined in C2C12/Mock and C2C12/Stab2 cells using quantitative real-time PCR. Data are presented as mean ± s.d. (n=3). (**c**) C2C12/Mock and C2C12/Stab2 cells were induced to differentiate for 24 or 48 h. Cells were then fixed and immunostained with anti-MyHC antibody. Representative microscopic fields are shown. Scale bar, 100 μm.

**Supplementary Figure 7**

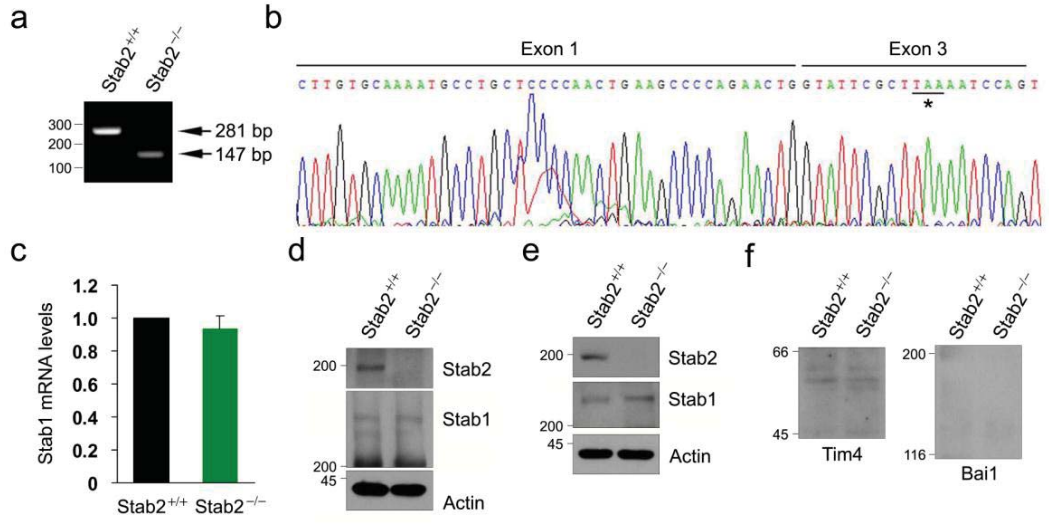


**Supplementary Fig. 7. Characterization of Stab2-deficient mice.** (**a,b**) Deletion of exon 2 in TA muscle of Stab2-deficient mice was confirmed by RT-PCR (a) and DNA sequencing of PCR product (b). Asterisk indicates stop codon. (**c**) Stabilin-1 mRNA levels were analysed in TA muscle of 9-week-old male *Stab2*$^{+/+}$ and *Stab2*$^{-/-}$ mice by quantitative real-time PCR (n=3). (**d,e**) Expression of stabilin receptors was analysed in TA muscle (d) and primary myoblasts (DM2, e) by immunoblotting. (**f**) Tim4 and Bai1 expression was analysed in TA muscle of 9-week-old male *Stab2*$^{+/+}$ and *Stab2*$^{-/-}$ mice by immunoblotting using anti-Tim4 (1:500) and anti-Bai1 (1:500) antibodies, respectively. Representative results are shown.

**Supplementary Figure 8**

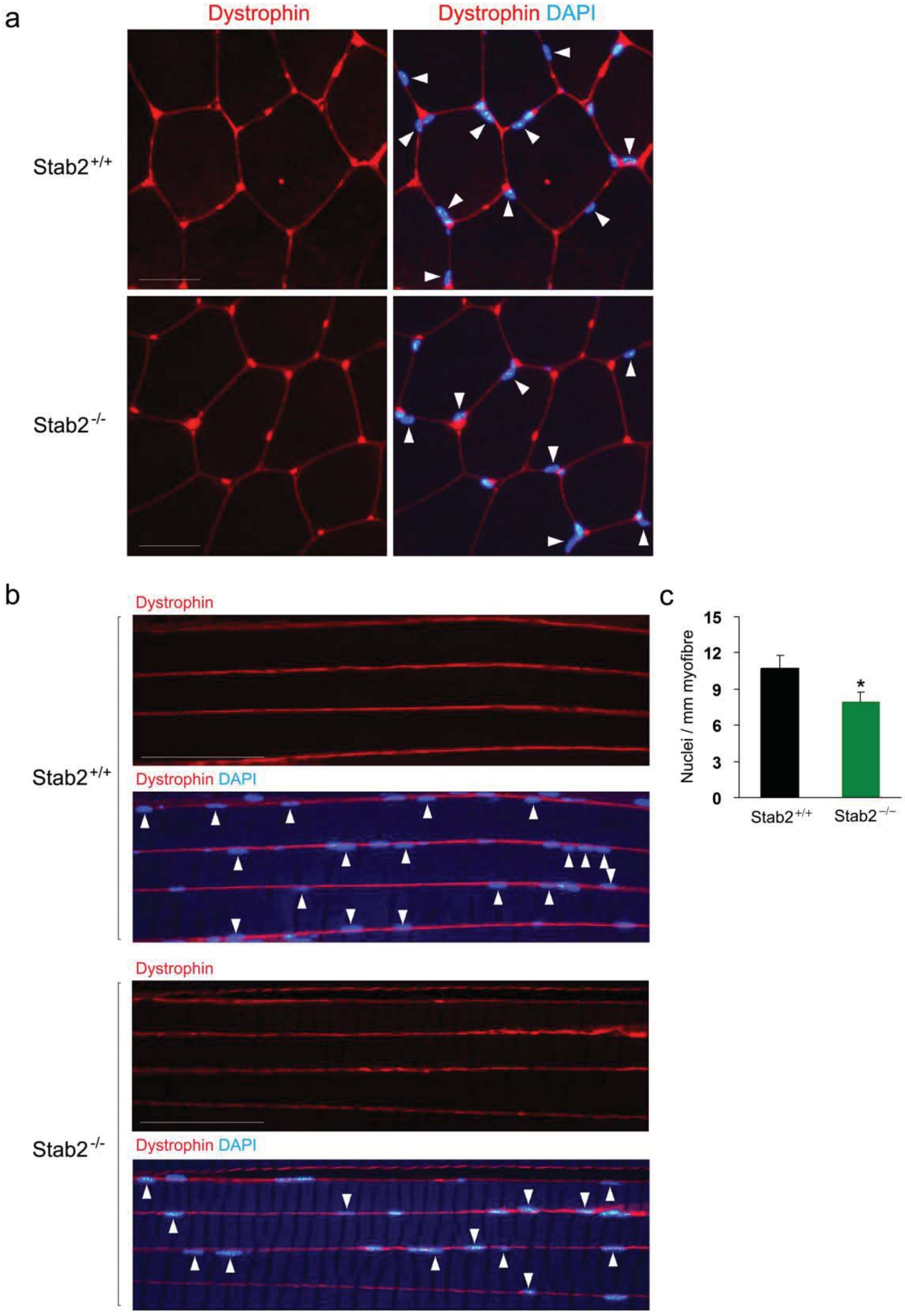

**Supplementary Fig. 8. Myonuclear numbers are reduced in *Stab2*$^{-/-}$ myofibres.** (**a**) Cross-sections of *Stab2*$^{+/+}$ and *Stab2*$^{-/-}$ TA muscles were stained with anti-dystrophin antibody (red) and DAPI (blue). Arrowheads indicate myonuclei within myofibres. Scale bar, 25 μm. (**b**) Longitudinal sections of *Stab2*$^{+/+}$ and *Stab2*$^{-/-}$ TA muscles were stained with anti-dystrophin antibody (red) and DAPI (blue). Arrowheads indicate myonuclei within myofibres. Scale bar, 100 μm. (**c**) The number of nuclei per mm of myofibre (longitudinal sections) was counted for TA muscles of *Stab2*$^{+/+}$ and *Stab2*$^{-/-}$ mice. Data are presented as mean ± s.d. (n=5) of each genotype. Asterisks indicate statistical significance (*$P < 0.05$, Student's *t*-test).

**Supplementary Figure 9**

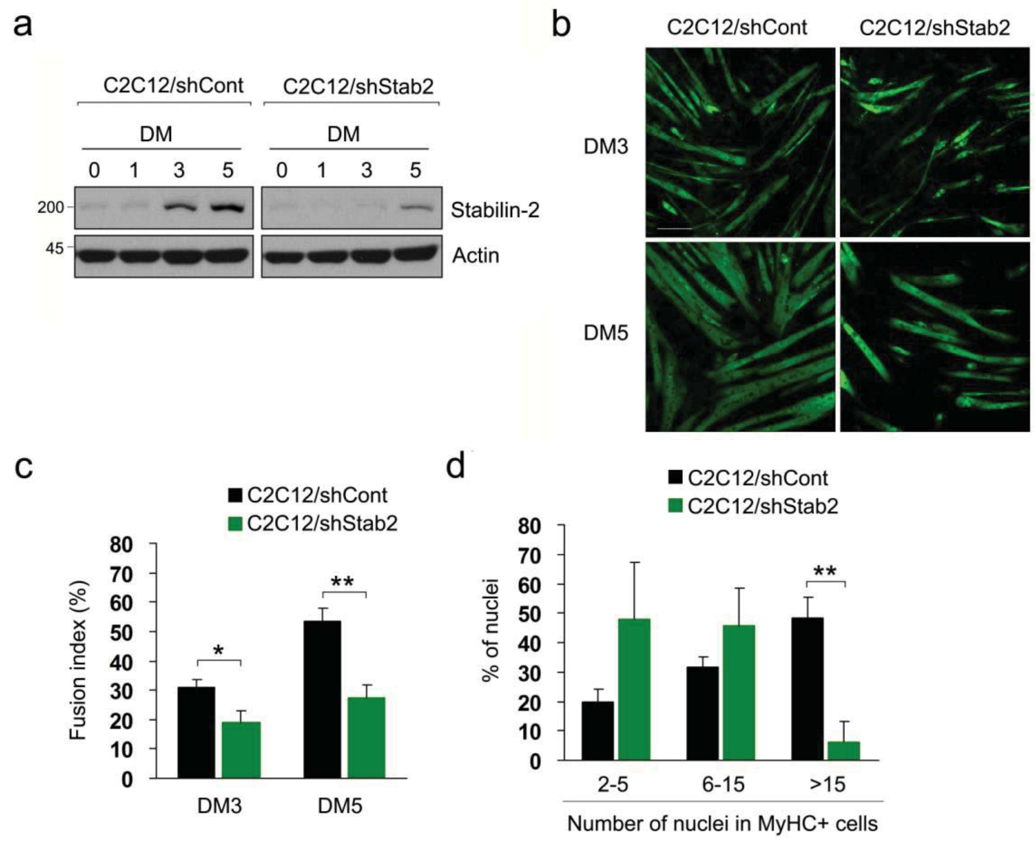


**Supplementary Fig. 9. Knockdown of stabilin-2 inhibits myotube formation during myogenic differentiation.** (**a**) C2C12 cells stably transfected with plasmid encoding shRNA against mouse stabilin-2 (C2C12/shStab2) or control shRNA (C2C12/shCont) were generated. Stabilin-2 expression was evaluated by immunoblotting with anti-stabilin-2 antibody. (**b**) Representative images of myotube formation in C2C12/shCont and C2C12/Stab2 cells after 3 and 5 days of differentiation (DM3 and DM5). Scale bar, 100 μm. (**c**) C2C12/shCont and C2C12/shStab2 cells were fixed and immunostained for MyHC after 3 or 5 days of differentiation, and fusion indices were determined. Data are presented as mean ± s.d. (n=3). Asterisks indicate statistical significance (*$P$ < 0.05, **$P$ < 0.01, Student's *t*-test). (**d**) After 5 days of differentiation (DM5), the percentage of nuclei present in MyHC-positive myotubes with the indicated number of nuclei were determined in C2C12/shCont and C2C12/shStab2 cells. Data are presented as mean ± s.d. (n=3). Asterisks indicate statistical significance (**$P$ < 0.01, Student's *t*-test).

**Supplementary Figure 10**

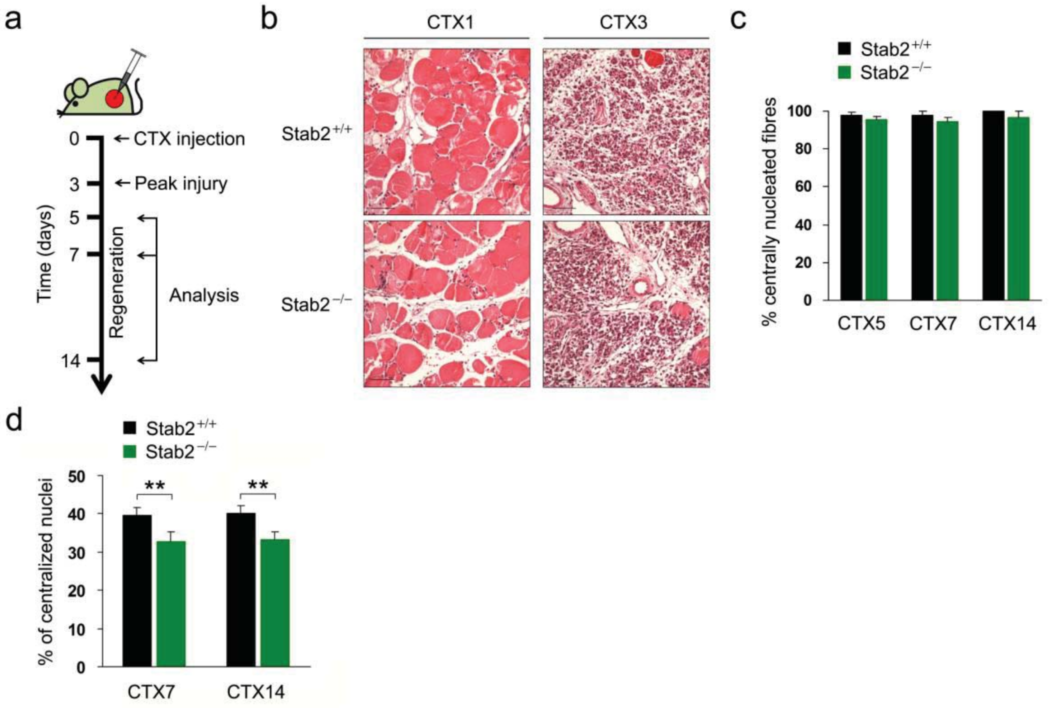


**Supplementary Fig. 10. Cardiotoxin injury is comparable for *Stab2*$^{+/+}$ and *Stab2*$^{-/-}$ muscles.** (**a**) Schematic diagram of the muscle regeneration experiments. (**b**) H&E staining of cross sections of *Stab2*$^{+/+}$ and *Stab2*$^{-/-}$ TA muscles at CTX1 and CTX3 (days 1 and 3 after CTX injection). Representative sections are shown. Scale bars, 50 μm. (**c**) The percentage of muscle fibres with centralized nuclei was quantified for *Stab2*$^{+/+}$ and *Stab2*$^{-/-}$ TA muscles at CTX5, CTX7, and CTX14. Data are presented as mean ± s.d. (n=5) of each genotype. (**d**) The percentage of centralized nuclei was quantified for Stab2$^{+/+}$ and Stab2$^{-/-}$ TA muscles at CTX7 and CTX14. Data are presented as mean ± s.d. (n=5) of each genotype. Asterisks indicate statistical significance (**$P$ < 0.01, Student's $t$-test).

**Supplementary Figure 11**

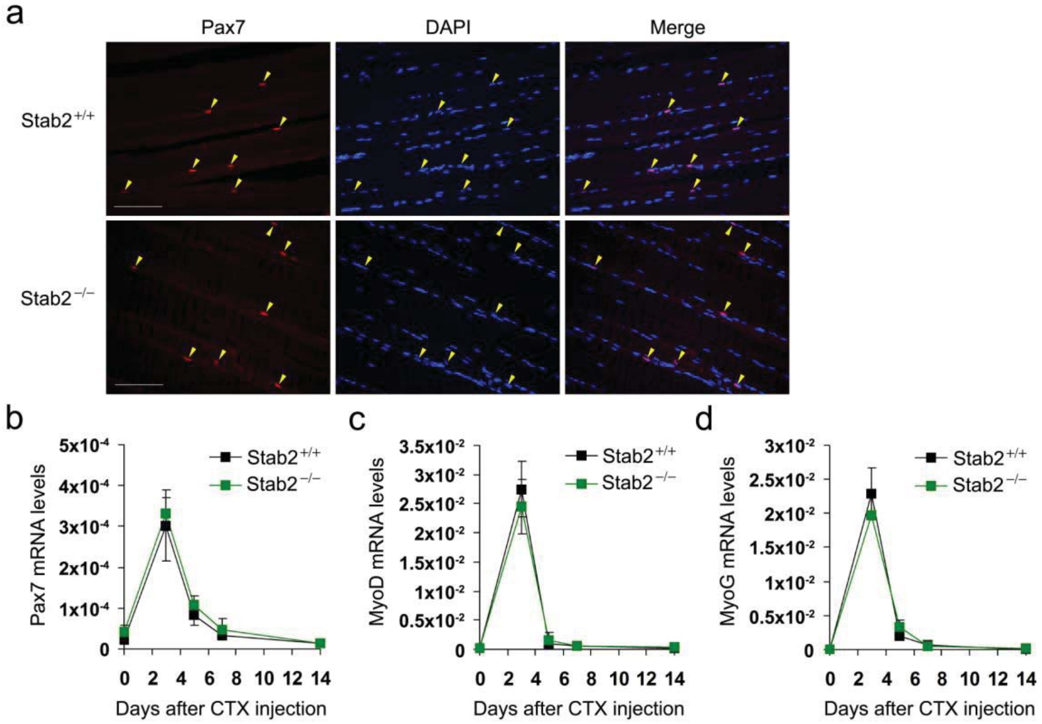


**Supplementary Fig. 11. Proliferation and activation of satellite cells is comparable for *Stab2*$^{+/+}$ and *Stab2*$^{-/-}$ muscles during muscle regeneration.** (**a**) Longitudinal muscle sections from TA muscle of 9-week-old male *Stab2*$^{+/+}$ and *Stab2*$^{-/-}$ mice were co-stained with pax7, a satellite cell specific marker, and DAPI to detect nuclei. Representative microscopic fields are shown. Arrowheads indicate pax7-positive satellite cells. Scale bars, 100 μm. (**b-d**) Expression levels of pax7, MyoD, and MyoG mRNA in TA muscle of wild type and Stab2-deficient mice were analysed during muscle regeneration after CTX injury. Data are presented as mean ± s.d. (n=3-5).

**Supplementary Figure 12**

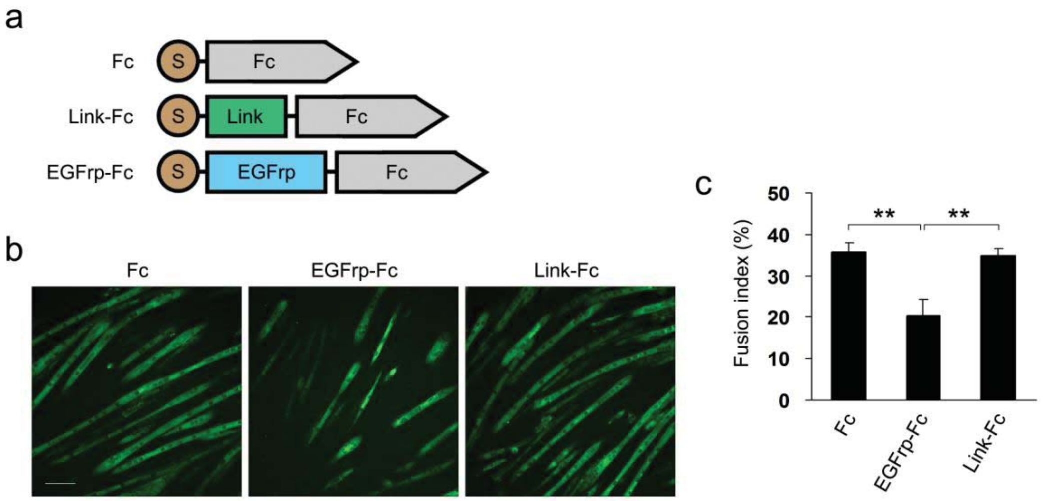


**Supplementary Fig. 12. Myoblast fusion was inhibited by masking PS in C2C12 cells using PS-binding domain of stabilin-2. (a)** Schematic diagrams of Fc-fusion proteins. (**b**) C2C12 cells were induced to differentiate in the presence of 20 μg $ml^{-1}$ of Fc (control), EGFrp-Fc (PS-binding domain of stabilin-2), or Link-Fc (hyaluronan-binding domain of stabilin-2) protein. After 5 days, cells were then fixed and immunostained with anti-MyHC antibody. Representative microscopic fields are shown. Scale bar, 100 μm. (**c**) At DM5, the fusion indices of C2C12 cells in the presence of Fc, EGFrp-Fc, or Link-Fc protein were calculated. Data are presented as mean ± s.d. (n=3). Asterisks indicate statistical significance (**$P$ < 0.01, Student's *t*-test).

**Supplementary Figure 13**

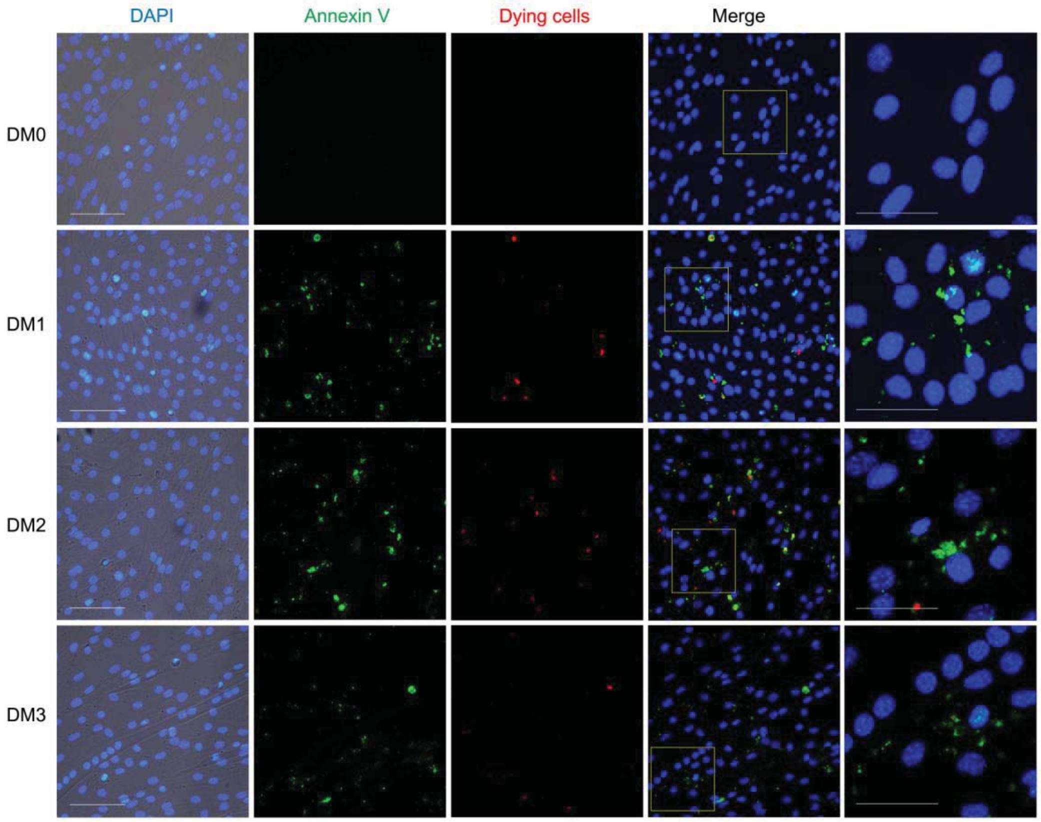


**Supplementary Fig. 13. Phosphatidylserine is expressed on the surface of healthy myoblasts.** C2C12 cells were induced to differentiate for the indicated times and stained with Alexa Fluor 488-conjugated Annexin V (for phosphatidylserine) and Fixable Viability Dye eFluor 660 (for dying cells). Cells were then fixed and stained with DAPI. Representative microscopic fields are shown. Scale bar, 100 μm. Yellow boxes are shown at higher magnification (Scale bar, 50 μm).

**Supplementary Figure 14**

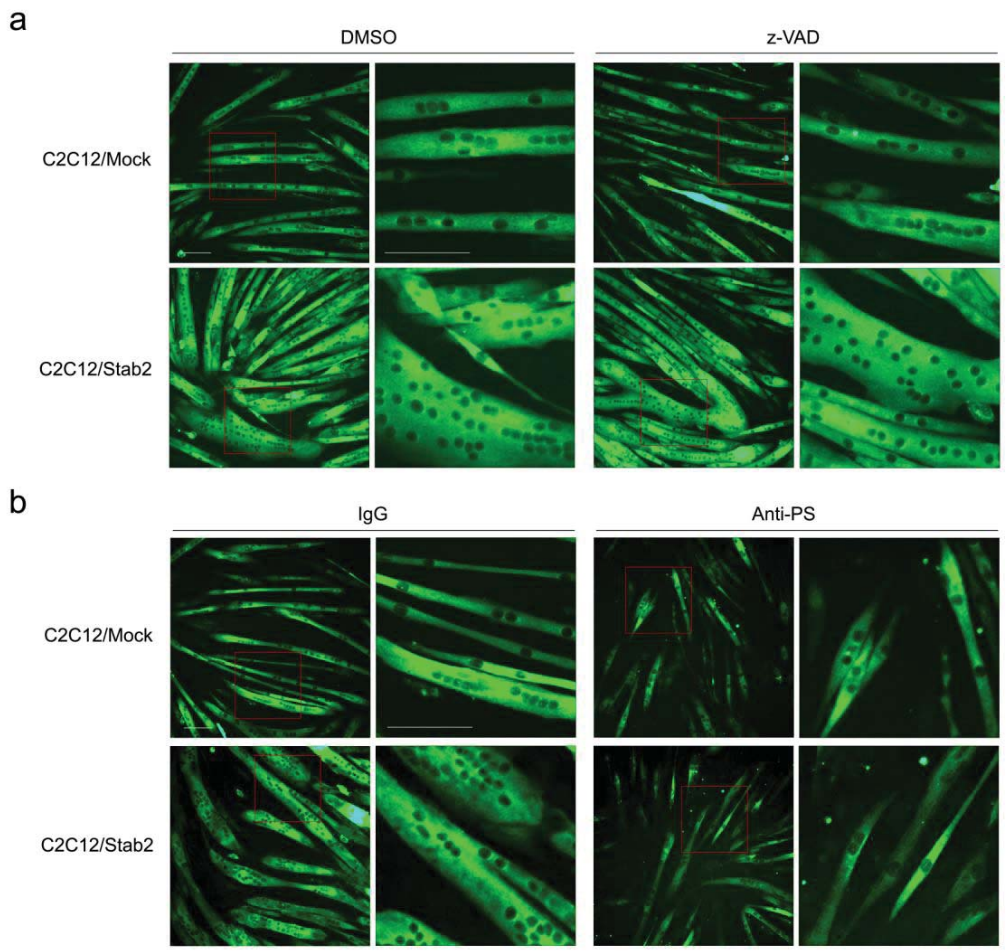


**Supplementary Fig. 14. Stabilin-2 mediates myoblast fusion in a PS-dependent manner.** (**a**) C2C12/Mock and C2C12/Stab2 cells were induced to differentiate for the indicated times in the presence of z-VAD-fmk (30 μM), a pan-caspase inhibitor, or DMSO. Cells were then fixed and immunostained with anti-MyHC antibody. Representative microscopic fields are shown. Scale bar, 100 μm. Red boxes are shown at higher magnification. (**b**) C2C12/Mock and C2C12/Stab2 cells were induced to differentiate for the indicated times in the presence of anti-PS antibody (10 μg $ml^{-1}$) or mouse IgG (10 μg $ml^{-1}$). Cells were then fixed and immunostained with anti-MyHC antibody. Representative microscopic fields are shown. Scale bar, 100 μm. Red boxes are shown at higher magnification.

**Supplementary Figure 15**

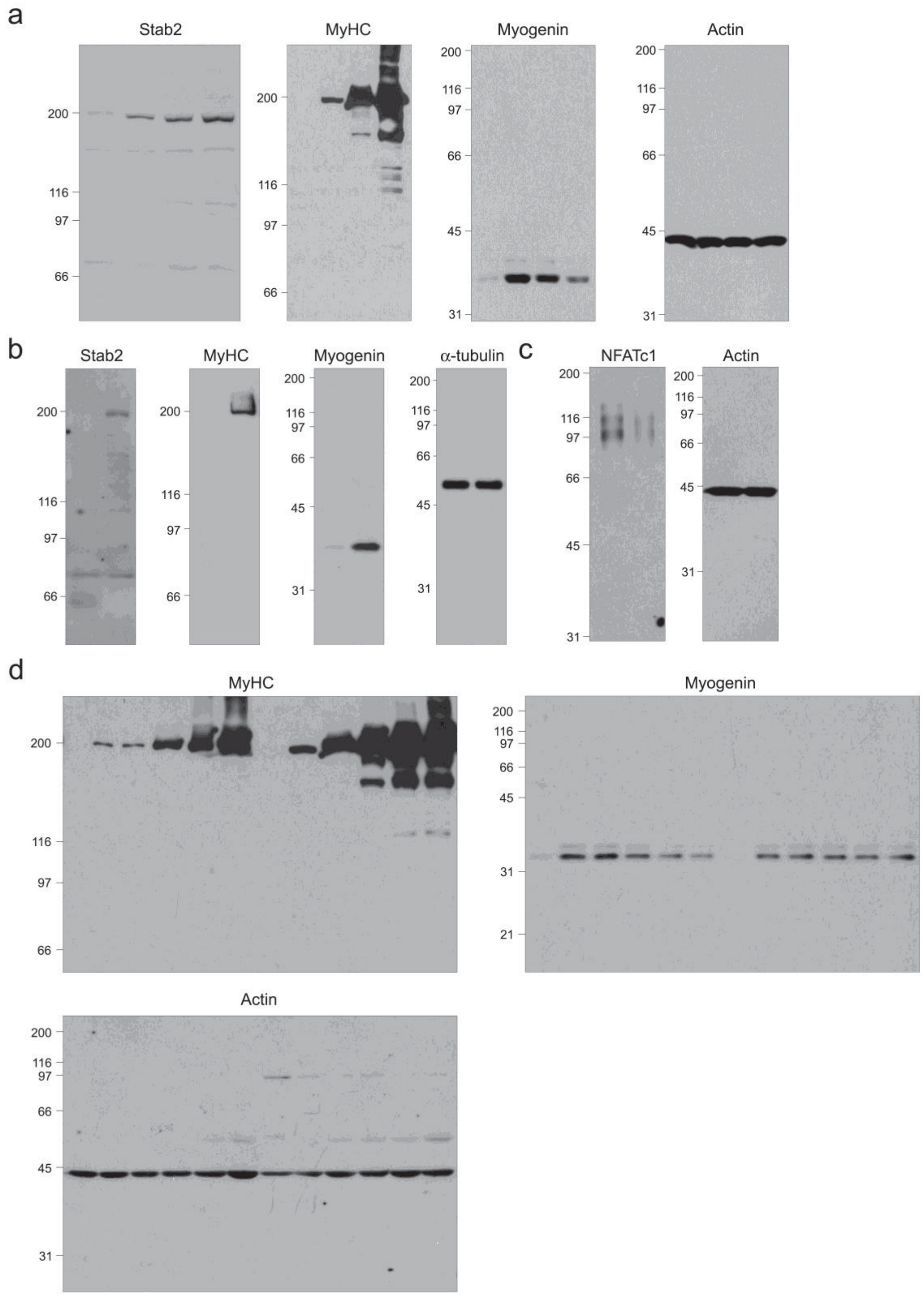


**Supplementary Fig. 15. Full-size of the blots presented in Fig. 1d (a), Fig. 1e (b), Fig. 2i (c), and Fig. 3f (d).**

**Supplementary Table 1**. Oligonucleotide sequences used in this study

Primers for RACE-PCR and promoter constructs

| Name | Sequence |
|---|---|
| Sense GSP | ATGGAACCTGTGAGTGCTACTCTGCG |
| Antisense GSP-1 | TCCCAGCTTACTCAGTGGCCAGGC |
| Antisense GSP-2 | CAGGCCCATCATATTTGCACACTGTAGAC |
| Antisense GSP-3 | AGTTAATTTGGCAGGGGTCCACAGGC |
| AAP | GGCCACGCGTCGACTAGTACGGGIIGGGIIGGGIIG |
| AUAP | GGCCACGCGTCGACTAGTAC |
| 5’ pStab2-1342 | AAAAGGTACCAGCTAAGCATTAGGCCT |
| 5’ pStab2-482 | AAAAGGTACCGATCTGGAACTTAAAGGG |
| 5’ pStab2-414 | AAAAGCTAGCAGAAGAAAGAATGAGTC |
| 5’ pStab2-182 | AAAAGCTAGCCATTGACAGGGCCGCAG |
| 5’ pStab2+83 | AAAAGCTAGCGGAAGGGATTTAAAAGT |
| 3’ pStab2 | AAAACTCGAGGAAATATTTGCTCCTTC |
| 3’ pStab2-mNFAT | AAAAGCTAGCCAACCACGCCATGGAAC |

Primers for quantitative real-time PCR

| Name | Forward | Reverse |
|---|---|---|
| Stab1 | TCCGCTGTACTCAAGGCTTCCA | CTTCTTGGCACAGGTGTAGGAAC |
| Stab2 | ATTGCTCTGGCTGCCTACTC | GTTGGCTGGCTTCTCACATC |
| h-Stab2 | ACTGGCTCCTTACCAAACCTGC | GAGCAAACACTGTGTAGGCATCG |
| Tim-1 | CTGGAATGGCACTGTGACATCC | GCAGATGCCAACATAGAAGCCC |
| Tim-4 | ATTCTCCCATCCACTTCACAG | CTATCTTCAGTGTTGTCTGGC |
| Bai1 | GCAGAACTGGACTTTGAGAAGCTTC | GTCTGGAGGTCAATGATGTC |
| Myh3 | ACCTCTAGCCGGATGGT | AATTGTCAGGAGCCACGAAAAT |
| Pax7 | CCATCAAGCCAGGAGACAGC | CCACAGGAAGAAGTCCCAGC |
| MyoD | AACTGCTCTGATGGCATGATG | TGGAGATGCGCTCCACTATG |
| MyoG | GCAAGGTGTGTAAGAGGAAG | TGTGGGAGTTGCATTCACTG |
| GAPDH | AACATCAAATGGGGTGAGGCC | GTTGTCATGGATGACCTTGGC |

Primers for Promoter enzyme immunoassay and EMSA

| Name | Sequence |
|---|---|
| NFAT-wt (sense) | Biotin-GTGGTTGTGGAAACATTGAC |
| NFAT-wt (antisense) | GTCAATGTTTCCACAACCAC |
| NFAT-mut (sense) | Biotin-GTGGTTGGCTAGCCATTGAC |
| NFAT-mut (antisense) | GTCAATGGCTAGCCAACCAC |

Primers for ChIP assay

| Name | Forward | Reverse |
|---|---|---|
| Stab2 promoter | TGGGACCGTGCTCACTTATG | CTCTAGTCCTGCCTACTGCTG |
| GAPDH | CTCTAGTCCTGCCTACTGCTG | ATGTAGGCCATGAGGTCCAC |

Primers for genotyping

| Name | Sequence |
|---|---|
| Stab2 (sense) | GGACACAGGCCATAGCAACTAA |
| Stab2 (antisense) | GGGTTTCTCACTGACCTGGA |
| Stab2 (Ex2-antisense) | GACTGGCACTCCGTCTTGAT |
| Stab2-Ex1 (sense) | GCTGCCTTTAATCCTCATCTTC |
| Stab2-Ex3 (antisense) | GCAGGTAATCCTTCCTACAG |

Primers for expression vector

| Name | Sequence |
|---|---|
| NFATc1 (sense) | AAAAAGGATCCGTCAGAGCGAGACTCAGAGG |
| NFATc1(antisense) | AAAAACTCGAGCACGCCACGCTGCTTTACGG |
| EGFrp (sense) | AAAAGGATCCGAGAAGAGGAGATGC |
| EGFrp (antisense) | AAAACTCGAGGCGGCAGGTAAATCCATC |
| Link (sense) | AAAAGGATCCACTGTTGGGGTGTTCCATCTAC |
| Link (antisense) | AAAACTCGAGCCGATAGCAGAAGACATCCCAC |
